# DNA‐Enzyme Hybrid Nanostructures: Functional Materials to Modulate Enzymatic Activity

**DOI:** 10.1002/smll.74185

**Published:** 2026-06-15

**Authors:** Manar Elnaggar, Amelie Heuer‐Jungemann

**Affiliations:** ^1^ Max Planck Institute of Biochemistry Center for Nanoscience LMU Munich Germany; ^2^ Faculty of Chemistry and Chemical Biology Research Center One Health Ruhr TU Dortmund University Dortmund Germany

**Keywords:** biocatalysis, DNA origami, DNA‐enzyme hybrid nanostructures, enzymes

## Abstract

DNA nanotechnology enables the precise assembly of complex and versatile 2‐dimensional (2D) and 3‐dimensional (3D) nanostructures. Coupling enzymes to such DNA nanostructures offers great control over the enzymes’ positioning and stoichiometry to modify their activity and unleash their maximum catalytic capacity. While enzymes hosted on DNA nanostructures have shown predominantly enhanced enzymatic activity, existing hypotheses such as proximity effects, electrostatic interactions, and pH modulation fail to fully account for the observed enhancement. Protection against enzymatic deactivation plays a role, but additional mechanisms, including potential hydration layer stabilization, require further investigation. Furthermore, spatial proximity alone does not guarantee efficiency in multi‐enzyme cascades, particularly in systems with unbalanced kinetics. Another big challenge remains when it comes to addressing disproportionally large enzymes. In this review, we summarize recent literature on DNA‐enzyme hybrid nanostructures and critically discuss the proposed hypotheses, aiming to shed light on the change in enzymatic activity upon conjugation to DNA nanostructures. We highlight gaps and remaining open questions in the field as well as giving a brief overview of potential applications of such DNA‐enzyme hybrid nanostructures.

## Graphical abstract

DNA–enzyme hybrid nanostructures enable precise spatial and stoichiometric control over enzyme organization, offering a powerful platform to modulate catalytic activity. This review critically evaluates key mechanistic hypotheses, including proximity effects, microenvironment changes, confinement, and stabilization, as well as highlighting inconsistencies in current understanding. It further outlines potential applications of such hybrid systems.

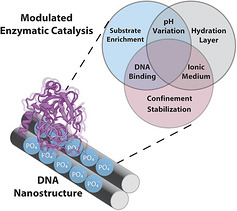

## 1 Introduction

The expanding field of synthetic biology, along with broader biotechnological applications, increasingly relies on enzymatic processes. Enhancing enzyme kinetics remains crucial, particularly when designing multistep cascade reactions, metabolic networks, or engineered synthetic cells. Notably, confining or controllably immobilizing enzymes on or in pre‐formed containers has emerged as a key strategy, consistently demonstrating significant enhancement of enzymatic performance [1, 2, 3]. Immobilization strategies include using a wide variety of materials, such as inorganic nanoparticles (NPs), e.g., silica [4], metal oxides like titanium dioxide, and metals such as gold [5, 6], or quantum dots [7]. Also, carbon‐based nanomaterials [8, 9] and different polymers [10, 11] offer many possibilities for hosting enzymes in industrial and medical applications. Different conventional systems have demonstrated clear advantages in terms of robustness, scalability, and cost‐efficiency. For example, porous inorganic supports and metal–organic frameworks (MOFs) provide high surface areas and protective microenvironments that enhance enzyme stability under harsh industrial conditions [12], while polymer‐based matrices offer straightforward upscale processing, reproducibility, and tunable mechanical properties [2]. Similarly, virus‐like particles (VLPs) enable encapsulation within biologically derived compartments, often improving enzyme stability and biocompatibility, and in some cases activity retention [13]. However, these platforms generally lack the level of spatial and numerical precision required to systematically study the effects of immobilization or confinement on enzymatic activity, highlighting the need for alternative approaches that enable more precise control.

**FIGURE 1:**
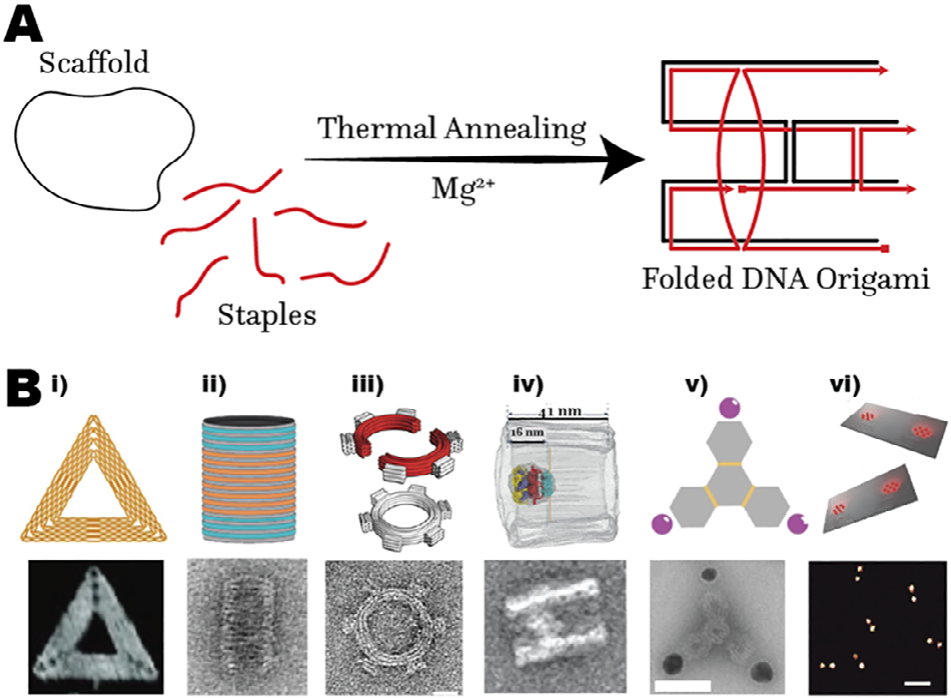
DNA origami synthesis and design: (A) Principle of DNA origami: a long ssDNA scaffold (black) is mixed with many short ssDNA staples (red) in a suitable Mg^+2^ containing buffer and annealed in a thermal cycler. Base pair hybridization of the staples to the scaffold brings distant sequences together to fold into pre‐designed shapes. (B) Examples of different DNA origamis designed with complex shapes: (i) triangular DNA origami [17], ii) barrel‐shaped DNA cage [18], (iii) 25 nm diameter gear with six‐tooth six‐helix bundle designed with bending [19], (iv) DNA cage hosting the unfolding p97 enzyme [20], (v) hexagonal modular DNA tetramer hosting gold NPs [21], (vi) rectangular DNA pegboard for hosting precisely located fluorophores [22]. (B‐i) Reproduced with permission [17]. Copyright 2019 MDPI. (B‐ii) Reproduced with permission [18]. Copyright 2020, Springer Nature. (B‐iii) Reproduced with permission [19]. Copyright 2009, AAAS. (B‐iv) Reproduced with permission [20]. Copyright 2024, Springer Nature. (B‐v) Reproduced with permission [21]. Copyright 2025, Springer Nature. (B‐vi) Reproduced with permission [22]. Copyright 2014, Springer Nature.

In recent years, advances in DNA nanotechnology have allowed the creation of complex structures to host and organize biomolecules with nanoscale addressability, controllable orientation, and programmable structural features. In the 1980s, Ned Seeman first proposed the design of immobile Holliday junctions formed from four simple single strands of DNA to create complex nanostructures [14]. Since then, many different DNA nanostructures have been synthesized, ranging from small structures to large crystal lattices [15]. Meanwhile, DNA origami emerged in 2006 as another very robust and highly efficient technique for the fabrication of a plethora of different DNA‐based nanostructures [16]. In this method, a long single‐stranded (ss) DNA scaffold (typically viral DNA of around 7500 nucleotides) is folded through complementary hybridization with hundreds of short synthetic ssDNA staples. These staples bring together distant parts of the scaffold to assemble the desired structure (Figure 1). Furthermore, these staples, when elongated, can be used as handles protruding out of the DNA origami to act as attachment sites for the precise conjugation of guest molecules such as e.g., proteins, NPs, or fluorescent dyes with nanometer (nm) precision [15].

For that reason, DNA nanostructures and in particular DNA origami present an excellent platform to host functional enzyme systems for biocatalysis. In contrast to conventional immobilization platforms such as MOFs, polymer matrices, or VLPs, DNA nanostructures uniquely enable nanometer‐scale spatial addressability and precise control over enzyme stoichiometry and positioning. They can act as a model for biochemical nanofactories that mimic the efficiency and harmony of biological systems. Within such synthetic systems, it is possible to modify the rate of certain chemical and biological reactions by providing unique control over the environmental variables affecting each component of the reaction of interest: e.g., different enzymes, substrates, and cofactors [23]. DNA‐enzyme hybrid nanostructures are a particular focus of attention owing to the great potential they promise in the field of biocatalysis, as it has been reported that many enzymes exhibit enhanced catalytic activity upon attachment to DNA and DNA nanostructures [24, 25, 26, 27].

An ever‐increasing interest in deploying DNA nanotechnology for enzyme immobilization and catalysis was sparked after Niemeyer and co‐workers [28] as well as Willner and co‐workers [29] first showed increased enzymatic activity of the enzyme pair glucose oxidase (GOx) and horseradish peroxidase (HRP) when placed in close proximity on a DNA duplex or DNA tile. Since then, numerous studies have explored this model system further to unravel the factors involved in the observed activity enhancement upon conjugation to DNA nanostructures. Many hypotheses emerged, including, among others: controlled proximity of enzymes to substrates, connected hydration layers, confinement and compartmentalization, control over the microenvironment around the enzyme, including local pH modification and substrate interaction with DNA, enzyme stabilization and protection against detrimental external factors. However, reported results often failed to confirm specific hypotheses and showed inconsistent reproducibility. Therefore, a deeper and more comprehensive examination of the intrinsic differences between each enzyme and the interplay between DNA and enzymes or substrates is required. Additionally, the experimental setup in each study must be carefully considered. These steps are essential to shed light on the underlying mechanisms and unlock the full potential of DNA‐enzyme hybrid nanosystems.

Earlier reviews have addressed this important topic; however, many are now several years old and require updating [30, 31, 32]. Others provide broad overviews of enzyme immobilization across diverse nanomaterials [33], rather than focusing specifically on nucleic acid‐based systems such as DNA nanostructures, while some are primarily methods‐oriented [34]. Consequently, there remains a clear need for a comprehensive, up‐to‐date, and critical review that consolidates recent advances and provides deeper mechanistic insight into enzyme behavior on DNA nanostructures. Here, we summarize recent literature on DNA‐enzyme nanostructure hybrids and critically discuss several of the proposed hypotheses aiming to explain the often observed change in enzymatic activity in such systems. We also give a brief outlook of potential applications of DNA‐enzyme hybrid nanostructure models.

## 2 Conjugation of Enzymes to DNA and DNA Nanostructures

In order to create DNA‐enzyme hybrid nanostructures, the strategy for conjugation requires careful consideration of multiple factors, including conjugation chemistry, protein functionality, and final design requirements (e.g., single or multiple attachment site(s), site specificity, etc.). To date, several approaches have been developed to attach enzymes, cofactors, and substrates to DNA nanostructures through either nonspecific or site‐specific interactions. These strategies leverage chemical modifications of nucleotides, site‐specific protein labeling, and bioorthogonal chemistries to achieve precise attachment while preserving biological activity [65, 66, 67]. Some of the most commonly employed strategies are outlined below. For a more detailed review on the topic of DNA–protein conjugation, the interested reader is referred to the following article [68].

**FIGURE 2:**
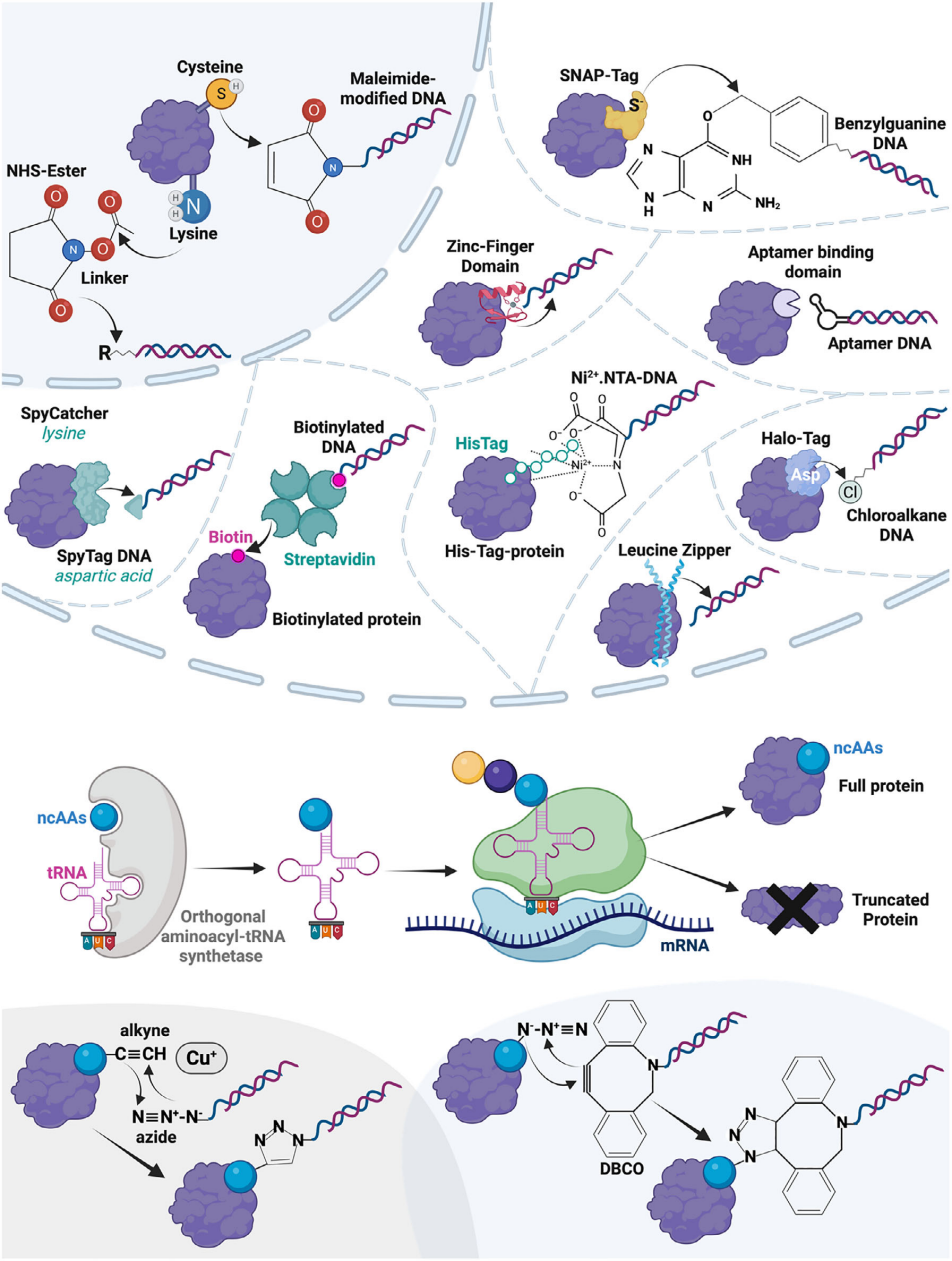
Overview of enzyme conjugation strategies to DNA strands for nanoscale spatial organization: (A) Nonspecific chemical conjugation methods target naturally occurring amino acid side chains. Maleimide‐functionalized oligonucleotides react with cysteine thiol groups, while NHS ester‐containing chemical linkers react with lysine groups to bind the enzyme to a modified oligo; depending on the linker used, the oligo could be ─SH or ─COOH modified. (B) Site‐specific tagging can rely on affinity interactions such as HisTag binding to Ni^2^^+^‐NTA‐modified DNA, biotin–streptavidin interactions with biotinylated DNA, and aptamer‐binding domains. Also, site‐specific labeling utilizes engineered protein domains that bind DNA with high specificity. Examples include covalent ligation via HaloTag and SNAP‐tag systems, or non‐covalent interactions via zinc fingers, leucine zippers, and SpyTag/SpyCatcher pairs. (C) Genetic code expansion enables the incorporation of ncAAs bearing bio‐orthogonal reactive groups into the protein of interest. This is achieved via an orthogonal tRNA–synthetase (gray) pair that incorporates non‐canonical amino acids (ncAAs) (blue) at specific codons (e.g., amber stop codon). These modified residues can then undergo click chemistry reactions for covalent DNA attachment. Figure created with BioRender.com.

One of the most widely used conjugation strategies for covalent modification of proteins with oligonucleotides involves the nonspecific targeting of natural reactive residues on the protein of interest, mainly cysteines or lysines. Depending on the residue, different linker molecules are used to create covalent bonds, providing robust and stable linkages. One common approach involves maleimide‐thiol chemistry. Thiol‐functionalized target molecules or accessible cysteines on proteins react with maleimide‐modified oligonucleotides to form stable thioether bonds [45, 69]. Another commonly employed strategy is N‐hydroxysuccinimide (NHS) ester chemistry, which facilitates the formation of amide bonds between lysine moieties and modified DNA strands via chemical linkers (Figure 2A). Targeting cysteine or lysine residues for chemical modification is simple, replicable, cost‐ and time‐efficient, with all reagents commercially available. Nevertheless, as lysine groups are often highly abundant on the surface of proteins, lysine NHS‐based conjugation has poorer site‐selectivity compared to cysteine functionalization [70]. Despite this limitation, lysine modification remains the most widely employed approach for enzyme conjugation in DNA nanostructure systems, owing to its synthetic simplicity and broad applicability across diverse proteins, as evidenced by numerous studies in the literature [20, 25, 26, 29, 36, 38, 49, 50, 51, 52, 54, 55, 56, 57, 62, 71].

However, there are other connection strategies which can offer much higher degrees of spatial and stoichiometric precision of conjugation. Site‐specific coupling methods include, among others: affinity‐based tags, sequence‐specific DNA‐binding tags, or covalent bonding to non‐canonical amino acids (ncAAs). Among the affinity‐based strategies, biotinylated proteins can be attached to biotinylated DNA nanostructures via (strept)avidin without modifying their intrinsic properties. The biotin–avidin interaction is the strongest affinity‐based interaction in nature [23, 72, 73] (Figure 2B). However, each streptavidin molecule can bind up to 4 biotin molecules, limiting the control over the number of interacting molecules. In addition, streptavidin's large size (MW 55 kD) may introduce steric hindrance to the nanostructure. Another affinity‐based strategy is the histidine tag (HisTag) metal‐assisted interaction with nitriloacetic acid (NTA) (Figure 2B). Many enzymes and proteins are commercially available with a HisTag, generally used for purification. NTA‐modified DNA is also readily available, making this an attractive approach [64].

Further, a wide range of DNA‐binding tags can be genetically fused to a protein of interest to facilitate attachment to DNA nanostructures (Figure 2B). Among many available tags, HaloTag [20, 42, 55, 57, 59, 60, 61, 63, 71], SnapTag [39, 58, 59, 60, 63], SpyTag [27, 74], zinc finger protein [39, 53, 58, 60], and leucine zippers [42, 53, 58, 60, 61] have been used to host enzyme molecules in DNA nanostructures (Figure 2B). Fusion proteins allow the biomolecule of interest to interact with the DNA without a direct chemical modification to maintain the native state. This is particularly interesting for hosted enzymes as it preserves the quaternary structure of the enzyme for optimal activity. However, the size of the fusion protein might introduce unwanted bulkiness and hindrance, which can affect the host enzyme's function. In addition, the tag binding affinity is of crucial importance to keep the integrity of the structure at low concentration. Instead of protein binders, aptamers can be employed for sequence‐specific binding [43], enabling precise localization of proteins on DNA scaffolds without the need for chemical modifications [41] (Figure 2B). In addition, dead Cas9 (dCas9)‐directed enzyme binding is another sequence‐specific DNA binding option. In this case, the protein of interest is bound to dCas 9, which is then directed to bind a DNA sequence specifically with the help of a single guide RNA [27, 46, 75, 76].

The most robust site‐selective and covalent conjugation strategy involves the use of click chemistry, which has emerged as a powerful tool for DNA–protein conjugation, offering bioorthogonal reactions that proceed efficiently under mild conditions with fast kinetics [77, 78, 79]. The toolbox of click reactions encompasses reactions such as the Staudinger ligation between azides and phosphines, the copper‐catalyzed azide–alkyne cycloaddition (CuAAC), strain‐promoted azide–alkyne cycloaddition (SPAAC), oxime and hydrazone ligations, strain‐promoted alkyne–nitrone cycloaddition (SPANC), tetrazine–trans‐cyclooctene ligation (Tz‐TCO), and quadricyclane ligation [77, 80]. The unique functional groups required for click chemistry can be incorporated into proteins using ncAAs [44, 81] (Figure 2C). Genetic code expansion enables the incorporation of such ncAAs at defined positions within proteins through engineered tRNA‐synthetase pairs, ensuring minimal disruption to protein structure and function [81]. Additionally, ncAAs with photocrosslinking moieties or reactive aldehydes further enhance the toolkit for protein immobilization [66, 67, 78, 79].

The choice of conjugation strategy strongly depends on the intended application, as each method presents trade‐offs in terms of stability, specificity, and biocompatibility. Covalent approaches ensure strong and irreversible attachment, making them ideal for long‐term studies and structural applications. In contrast, non‐covalent interactions offer dynamic and near‐native assembly, which is beneficial for maintaining the integrity of functionalized enzymes. Further, the mode of immobilization can significantly influence enzyme activity. For example, it was shown that SpyCatcher‐modified Lactobacillus alcohol dehydrogenase (LbADH) exhibited a 40% increase in activity compared to the free enzyme before immobilization on a DNA nanostructure [63]. In another study, SNAP‐tagged antifreeze protein attached to gold NPs resulted in a strong antifreeze‐active nanomaterial. However, HisTag‐captured antifreeze protein resulted in no activity at all [82]. These examples underscore the importance of thoroughly characterizing individual enzymes prior to further use.

## 3 Hypotheses for Observed Enzymatic Activity Enhancement in DNA–Enzyme Hybrid Nanosystems

A substantial body of work has explored the modulation of enzymatic activity upon conjugation to DNA and DNA nanostructures. These studies span both single‐enzyme systems and multienzyme cascades assembled on DNA nanostructures. While the influence of the DNA microenvironment on catalytic behavior appears to be broadly conserved for individual enzymes—whether operating independently or within a cascade—the role of spatial proximity becomes uniquely significant in multienzyme configurations. In such systems, the controlled co‐localization of sequential catalytic components enables mechanistic interrogation of proximity effects that cannot be captured in isolated enzyme studies. Notably, numerous reports demonstrate that cascade reactions proceed with enhanced efficiency when enzymes are tethered in close proximity on DNA nanostructures, as compared to their freely diffusing counterparts. A comprehensive overview of studies investigating both single‐ and multienzyme systems across diverse DNA nanostructures is provided in Table 1.

**TABLE 1:** Summary table of DNA‐enzyme hybrid nanostructures: The table compiles 35 representative studies, of which 19 (>50%) investigate enzymes on open DNA nanostructures without confinement (e.g., 1LS, DNA tiles, triangular structures). Among single‐enzyme systems, HRP is reported in five studies, while in multienzyme cascades, the GOx/HRP cascade appears in eight studies, making these the most extensively explored systems. Lysine‐based conjugation is the most frequently employed strategy (≥17 studies, ∼50%), followed by site‐specific approaches such as HaloTag and SNAP‐tag.

Enzyme(s)	DNA nanostructure	Enzyme coupling	Reported results				Findings and comments	Refs.
			Total activity	Affinity (K_m_)	Turnover rate (K_cat_)	Catalytic efficiency (K_cat_/K_m_)	·	
**Single‐enzyme systems**								
**HRP**	DNA tiles	Encapsulation by mixing it with a DNA tile cage using droplet microfluidics	Arbitrary increase (not numerically reported)	—	—	—	·The design enables temperature‐controlled open/close transition (open at 37°C, closed at 4°C); this allows HRP loading/release at 37°C and retention in the cavity at 4°C, conformational switching validated by electrophoretic mobility shift and FRET ·Fluorescently labeled HRP could not be encapsulated	[35]
**HRP**	DNA tiles	Lysine modification using sulfo‐SMCC	—	∼2.9 and ∼2.4‐fold K_m_ decrease for TMB and para‐aminophenol, respectively	Statistically indifferent	—	·HRP modified with DNA tiles to tune catalytic activity ·Studied substrate binding and residence time near active site ·Enhanced kinetics attributed to van der Waals‐like interactions ·Effect explained by increased local substrate concentration near DNA scaffold	[25]
**HRP**	DNA origami nanocapsule with open/close mechanism	Lysine modification using sulfo‐SMCC	1.5 and 1.8‐fold higher in the closed and open state, respectively, compared to free HRP	Statistically indistinguishable between open and closed state	—	—	·Reversible opening/closing via pH changes ·Mechanism: 8 pH‐responsive triplex latches (hairpin + ssDNA) ·Opening/closing threshold tunable via TAT triplet content in latches ·HRP used as model protein under physiological conditions (150 mM NaCl, no MgCl_2_, blood plasma)	[36]
**HRP**	DNA cage	Lysine modification using sulfo‐SMCC	Approximately fivefold decrease in V_max _	Insignificant change	—	—	·DNA cage is encapsulated with capsid coating for protection ·HRP activity decreases upon encapsulation in DNA cage; decrease is further exacerbated after capsid coating ·Different substrates used: oPD, TMB, ABTS; only ABTS showed decreased affinity ·Enzymes were also tested at 10 nm vs. 100 nm inter‐enzyme distances, with no decrease in the total activity at longer distance, excluding proximity and substrate channeling effects	[37]
**αCh**	DNA origami nanocapsule with open/close mechanism	Lysine modification using BCL‐014	Threefold higher in open vs. closed state	—	—	—	·Reversible opening/closing via strand displacement (∼30% folding yield; α‐Ch encapsulation efficiency ∼5–10%) ·Enzyme activity losses: −42% after azide modification, additional −22% after ssDNA interaction ·Casein (fluorescent) better substrate than sAAPFpNA ·Reduced activity in closed state attributed to shielding + local pH effects	[38]
**CA or XR**	1LS	Fused to Zinc Finger‐SNAP Tag	∼1.3‐fold increase in packed vs. dispersed state for p‐NPA substrate. ∼1.5–1.6‐fold increase for more hydrophobic substrates (p‐NPB, p‐NPV)	—	—	—	·1LS used to position enzyme in packed or dispersed state ·Each state accommodates up to 4 enzymes ·Packed state shows faster reactions than dispersed state and higher inhibitor tolerance ·Effect is more pronounced for hydrophobic substrates ·Activity enhancement is independent of enzyme orientation or structural stability; it is also attributed to entropic water effects and increasing local substrate concentration in confined spaces	[39]
**CA or XR**	DNA origami nanocapsule with open/close mechanism	Fused to Zinc Finger‐SNAP Tag	Varied according to the substrate	—	—	—	·XR activity was assessed using a variety of substrates: D‐xylose, L‐glyceraldehyde, 4‐nitrobenzaldehyde, ethylbenzoylformate and o‐chloroacetophenone ·Change in activity according to the respective substrate (in order as above): 1.6, 1.5, 1.3, 0.85 and 0.79. This decrease in activity is in correlation with the substrate hydrophobicity. ·CA activity was assessed using p‐NPA, p‐NPB and p‐NPV. ·Enhanced activity only with p‐NPA ·Mechanistic study shows more hydrophilic substrates to reside closer to the DNA surface, meanwhile hydrophobic substrates reach closer to the enzyme active site	[40]
**α‐Thrombin**	Two DNA nanostructures: monolayer frame or bilayer cube	Affinity binding via thrombin‐binding aptamer	—	Approximately threefold decrease in K_m_ upon attachment to DNA nanostructures	∼1.5‐fold increase upon attachment to DNA nanostructures	∼4‐6‐fold increase	·Reaction rates increase in the presence of DNA, independent of substrate type ·The effect depends on substrate charge ·Neutral and negatively charged substrates show faster cleavage, likely due to transition state stabilization ·Positively charged substrates show reduced activity	[41]
**XDH**	Dimeric hexagonal DNA prism with Open/Close mechanism	Fused to the basic Leucine Zipper protein GCN4‐HaloTag	Statistically indistinguishable between open and closed state	—	—	—	·Reversible opening/closing via strand displacement (∼90% folding/closing yield at 1:1 scaffold: linker ratio); higher linker ratio (1:10) reduced yield to ∼61% ·XDH activity enhanced in closed state ·Enhancement attributed to ordered hydration layer near DNA surface ·Likely effects: enzyme stabilization and/or increased local concentration of hydrophilic substrates	[42]
**Trypsin**	Triangular DNA origami	Trypsin–ligand non‐covalent binding on the DNA surface	—	—	—	—	·DNA origami used as platform to study protein–ligand interactions (trypsin) ·Observed nonspecific binding of trypsin to DNA origami ·AFM resolution insufficient to distinguish mono‐ vs. bidentate binding	[43]
**Caspase 9**	1LS	N‐terminus modified with unnatural amino acid p‐azidophenylalanine	∼23‐fold increase	Slightly higher K_m_ on DNA origami	—	—	·1LS used as synthetic apoptosome platform to study proximity‐induced protein–protein interactions ·Caspase‐9 monomers colocalized to induce dimerisation ·Inter‐monomer distances varied from 6 to 36 nm ·Enzyme incorporation efficiency ∼75% per handle ·Highest activity seen at shortest distance (6 nm) yielding ∼23‐fold activation ·Activity linked to dimerisation requirement of caspase‐9 ·DNA‐bound dimers show increased resistance to inhibitors ·Activity increases with higher monomer density per structure ·DNA‐related effects (e.g., local pH) ruled out	[44]
**M.TaqI**	1LS	Native cysteine (C48S) mutated and another cysteine grafted at the C‐terminus, then linked via GMBS	—	—	—	—	·M.TaqI used to covalently transfer a biotin‐containing group onto target DNA at TCGA site ·Double anchoring intended to enhance enzyme–substrate interaction, yielding ∼37% of target modification vs. theoretical maximum ·Demonstrates feasibility, but requires further optimization	[45]
**Cas9**	Ring‐shaped DNA origami	Cas9/sgRNA attached to complementary DNA	DNA cleavage measured as 0% (caged), 46% (released), 74% (free)	—	—	—	·Cas9 attached via photocleavable (PC) linker for UV‐triggered release ·Maximum of ∼6 Cas9 units accommodated per nanoring ·Enables spatiotemporal control of genome editing: suppressed activity in confinement and partially restored upon release ·Potential for photo‐targeted therapy, but limited by UV safety concerns	[46]
**HRP**	Fully enclosed DNA nanocage	Lysine modification using sulfo‐SMCC	—	Insignificant change	Approximately ninefold increase	—	·Enzymes were selected in ascending molecular weight order: HRP 44 kDa, MDH 70 kDa, G6PDH 100 kDa, LDH 140 kDa, GOx 160 kDa, and *ß‐*Gal 450 kDa. ·Enzymatic activity enhancement in DNA nanocage reduces with size. ·Enhancement is likely due to the polyanionic and hydrated microenvironment, not just the confinement (tested with high conc. of NaCl), as well as stabilization of the active conformational state ·Most enzymes suffered decreased substrate affinity upon encapsulation.	[47]
**MDH**			—	∼1.5‐fold increase in K_m_	Approximately ninefold increase	—	·	
**G6PDH**			—	∼1.3‐fold increase in K_m_	Approximately fourfold increase	—	·	
**LDH**			—	∼2.4‐fold increase in K_m_	Approximately fourfold increase	—	·	
**GOx**			—	∼0.48‐fold decrease in K_m_	∼5.4‐fold increase	—	·	
***ß‐*Gal**			—	∼1.6‐fold increase in K_m_	∼0.19‐fold decrease	—	·	
**RuBisCO**	Dimeric hexagonal DNA prism with open/close mechanism	Fused to the modular dual adaptor CLIP–GCN4	Statistically indistinguishable between open and closed state	—	—	—	·Prism design allows confinement and crowding‐like effect ·Average loading: ∼6 RuBisCO dimers per nanostructure ·RuBisCO is tested in solvent‐exposed (open) vs. confined (closed) states ·Indicates spatial confinement and increased enzyme loading alone do not improve RuBisCO catalysis ·Suggests counteracting effects (electrostatic repulsion, conformational restriction) offset potential benefits	[48]
**Multi‐enzyme cascade systems**								
**GOx**	Hexagonal DNA tiles	Lysine modification using sulfo‐EMCS	Max Abs from 0.005 to 0.08 (∼16‐fold) (not numerically reported)	—	—	—	·Structures tested independently for GOx/HRP and GDH/NAD^+^ systems ·Reaction efficiency depends on inter‐enzyme distance ·Overall DNA origami geometry influences activity ·No direct energy/electron transfer through DNA scaffold	[29]
**HRP**				—	—	—	·	
**GOx**	1LS	Lysine modification using SPDP	∼15‐fold increase	—	—	—	·GOx/HRP were positioned at 10, 20, 45, or 65 nm ·Highest activity observed at 10 nm separation ·Co‐assembly yield is lowest at 10 nm due to steric hindrance ·Enhanced activity attributed to restricted Brownian motion ·Close proximity enables interaction via shared hydration shell	[49]
**HRP**				—	—	—	·	
**GOx**	1LS and rolled 1LS as short or long nanotube	Lysine modification using sulfo‐EMCS	Max Abs from 0.01 to 0.055 (∼5.5‐fold for the planar structure) and from 0.01 to 0.09 (approximately ninefold for the tubular structure (not numerically reported)	—	—	—	·GOx/HRP positioned at fixed 15 nm distance on planar scaffold and inside nanotubes (diameter ∼20 nm) ·Two fabrication methods tested: single‐step vs. stepwise assembly ·Short nanotubes improved substrate confinement, reducing diffusion to surroundings ·Enhanced reaction efficiency observed in nanotube‐confined system	[50]
**HRP**				—	—	—	·	
**GOx**	Hexagonal DNA cage	Biotin‐NeutrAvidin‐biotinylated HRP and GOx	Graphically represented (not numerically reported)	—	—	—	·Each monomer was loaded with either GOx or HRP ·Monomers assembled into dimeric tubular nanofactories via sequential process ·Significant activity enhancement observed in dimers	[23]
**HRP**				—	—	—	·	
**GOx**	DNA octahedron	Lysine modification using sulfo‐EMCS	∼2‐ or 1.5‐fold increase in activity upon attaching GOx/HRP colocalized on same octahedron vs. the free enzymes or the GOx/HRP attached each to separate octahedron, respectively	∼20%–25% decrease in K_m_ upon attaching GOx/HRP colocalized on same octahedron	∼2‐ or 1.5‐fold increase upon attaching GOx/HRP colocalized on the same octahedron vs. the free enzymes or the GOx/HRP attached each to separate octahedron, respectively	∼2.8‐ or 1.8‐fold increase upon attaching GOx/HRP colocalized on the same octahedron vs. the free enzymes or the GOx/HRP attached each to separate octahedron, respectively	·DNA wireframe octahedron used to build library of GOx/HRP arrangements to test enzyme orientation, spacing, and scaffold structure ·Lysine modification led to 10% and 40% activity loss in GOx and HRP, respectively. ·DNA sequence composition negligible for activity ·Higher activity at <10 nm spacing (colocalization effect) ·Activity variations linked to structural discontinuities in scaffold	[51]
**HRP**							·	
**GOx**	—Chemical linker (No DNA)	Lysine modification using SPDP	Statistically indistinguishable between free and conjugated cascade	Statistically indistinguishable between free and conjugated cascade	Statistically indistinguishable between free and conjugated cascade	—	·GOx chemically conjugated to HRP to mimic proximity without DNA origami scaffold ·Surface pH estimated using Poisson–Boltzmann equation (DNA surface potential ∼ −130 mV) ·Activity enhancement attributed to locally reduced pH near negatively charged DNA surface ·DNA scaffold creates a more optimal microenvironment for enzyme activity ·Modelling shows ∼1 pH unit decrease within ∼2 nm from DNA surface vs. bulk ·Rapid H_2_O_2_ diffusion in bulk indicates proximity effects are negligible ·Experimental results align with theoretical modelling	[52]
**HRP**		HRP Lysine groups were conjugated to modified‐GOx using maleimide chemistry (sulfo‐SMCC).				—	·	
**XR**	1LS	Fused to Zinc Finger‐SNAP Tag	—	—	∼9‐, 6.7‐ and 4‐fold increase upon placement of enzymes at 10, 54 and 98 nm distance	—	·1LS with three cavities designed to position enzymes at varying distances ·Target: 4 molecules of each enzyme per cavity, but XDH anchoring was not reproducible ·Enzyme positioning via ZF and LZ DNA‐binding proteins ·Shorter inter‐enzyme distances performed best ·Simulations indicate Brownian diffusion of intermediates between enzymes ·Mutant XR exhibits approximately twofold higher substrate affinity compared to native XR	[53]
**XDH**		Fused to the basic Leucine Zipper protein GCN4‐HaloTag	—	—		—	·	
**G6PDH**	DNA tiles	Lysine modification using SPDP	90‐fold enhancement for the shortest distance (7 nm), and up to 277‐fold upon adding more NAD^+^ arms	—	—	—	·DNA tiles used to localize G6PDH/MDH with a polyT20 “swinging arm” functionalized with NAD^+^ ·Enzymes positioned at 7, 14, or 21 nm from the swinging arm ·Shorter distances show higher reaction performance ·Multiple NAD^+^ near enzymes further improved activity ·G6PDH activity increased up to fourfold ·MDH activity increased up to twofold	[54]
**MDH**				—	—	—	·	
**MDH**	Three‐point star DNA nanostructure	Fused to HaloTag.	Fivefold increase in enzymatic output	—	—	—	·The three enzymes positioned on a three‐point star DNA structure (∼12 nm spacing) ·Geometry found to be more critical than distance for cascade efficiency ·Design promotes continuous intermediate depletion, driving the reaction forward	[55]
**OAD**		OAD and LDH were lysine‐modified using SPDP		—	—	—	·	
**LDH**				—	—	—	·	
**AGO**	Tetrahedral DNA nanostructure	Lysine modification using SPDP	∼5.9‐ and 7.7‐fold increase for the GOx/HRP and AGO/GOx/HRP enzyme systems, respectively	—	—	—	·Cascade activity enhanced from 1D < 2D < 3D architectures ·Enhancement is driven by reduced diffusion distances, higher local enzyme concentration, and more efficient intermediate transfer ·Overall cascade rate is governed by the slowest (rate‐limiting) enzyme (AGO) rather than proximity alone	[56]
**GOx**				—	—	—	·	
**HRP**				—	—	—	·	
**G6PDH**	1LS	G6PDH and MDH were fused to HaloTag	Five‐ to sevenfold increased enzymatic activity.	—	—	—	·Three‐enzyme cascade on 1LS enabling NAD^+^ substrate channeling ·Four‐arm Holliday junction used: one arm anchors to scaffold, opposite arm carries NAD^+^, remaining arms bind enzyme‐positioning anchors ·Directional control of NAD^+^ achieved via toehold‐mediated strand displacement ·Shorter NAD^+^ linker length shows higher activity ·Confirms importance of inter‐enzyme distance on system performance	[57]
**MDH**				—	—	—	·	
**LDH**		LDH was lysine modified using SPDP		—	—	—	·	
**CA**	1LS	Fused to Zinc Finger‐SNAP Tag	∼25%–40% decrease in total activity (estimated from curves)	—	—	—	·RuBisCO/CA co‐assembled on rectangular DNA sheet (96 × 100 nm) ·DNA sheet design: 4 corner sites for RuBisCO, 1 central site for CA (up to 4 CA) ·Average loading: ∼6.4 RuBisCO and ∼2.6 CA per sheet ·∼20 nm distance between CA and RuBisCO ·Co‐assembly resulted in reduced RuBisCO activity ·Attributed to preferential CO_2_ dehydration by CA, limiting RuBisCO carboxylation ·Suggests inter‐enzyme distance not dominant factor in this system ·Form II RuBisCO (Rhodospirillum rubrum) was used for simplicity	[58]
**RuBisCO**		Fused to the C‐terminal of the homodimer DNA‐binding protein GCN4		—	—	—	·	
**ADH**	1LS	Fused to HaloTag	∼1.6‐fold increased activity	—	—	—	·The focus is on re‐engineering SNAP_R5 by mutating 5 amino acids, achieving up to 11‐fold higher coupling efficiency to benzylguanine‐oligonucleotide ·Resulting in enzyme occupancy up to ∼75%	[59]
**ICDH**		Fused to SNAP Tag		—	—	—	·	
**XR**	Square pyramidal DNA nanostructure	Fused to Zinc Finger‐SNAP Tag	—	—	Approximately fourfold increase	—	·Activity tested in presence of BSA to assess nonspecific adsorption ·DNA origami provides protection against enzyme hydrolysis ·Both XR (preferential pH 6) and XDH (preferential pH 8) show enhanced activity on DNA ·Measured local pH shift ∼0.8 units near DNA surface is insufficient to explain enhancement for both enzymes ·Substrates are uncharged, and cofactor is near‐neutral; no significant local up concentration effects ·Suggests activity enhancement arises from factors other than pH or substrate enrichment	[60]
**XDH**		Fused to the basic leucine zipper protein GCN4‐HaloTag	—	—	> 4‐fold increase	—	·	
**XDH**	Hexagonal DNA prism with Open/Close mechanism	Fused to the basic leucine zipper protein GCN4‐HaloTag	Statistically indistinguishable between different distances or open/closed states	Kinetic parameters were not assessed after binding to the DNA nanostructure; however, absolute values of each enzyme were measured, showing XK to be ∼200‐fold faster with ∼600‐fold stronger substrate affinity		—	·Overall cascade is controlled by the slow upstream enzyme (XDH), and not by distance, channeling, or proximity ·No clear correlation between activity and inter‐enzyme distance ·Lack of enhancement attributed to imbalanced catalytic rates ·Downstream enzyme much faster, masking spatial effects on cascade performance ·Enzymatic activity was not assessed compared to free enzymes in the bulk solution	[61]
**XK**		Fused to the modular dual adaptor AZ Zinc Finger‐CLIP Tag					·	
**Pyruvate kinase (type II)**	1LS	Lysine modification using SPDP	∼2–2.5‐fold increase with ATP enrichment at DNA surface compared to the same enzymes/1LS assembly without aptamers	Approximately threefold decrease and approximately twofold increase in K_m_ at aptamer enrichment vs blocking, respectively	—	—	·1LS modified with 14 aptamers to enrich ATP near immobilized enzymes (∼45 nm spacing) ·Aptamer blocking reduces cascade activity (loss of ATP binding) ·Assembly yield ∼80%–94% ·Local ADP enrichment measured at ∼2.9‐fold ·Vmax remained unchanged ·Enhancement attributed to local substrate enrichment via aptamers, not directly via DNA nanostructure	[62]
**Hexokinase **					—	—	·	
**ADH**	1LS assembled on microbeads	Fused to HaloTag	Attachment to the 1LS DNA on microbeads shows 100% enhancement	—	Attachment to the 1LS DNA had insignificant effect; however, 1LS DNA on microbeads shows 122% enhancement in ADH K_cat_	—	·System compares nanoscale vs. microscale environment ·Nanoscale compartmentalization has weak effect on enzymatic output related to enzyme distance or reduced diffusion barriers ·Microscale confinement has stronger impact on cascade efficiency ·Highlights importance of larger‐scale spatial confinement over nanoscale arrangement, which can lead to substrate enrichment at larger scale ·ICDH is the rate‐limiting enzyme in the cascade	[63]
**ICDH**		Fused to SNAP Tag		—		—	·	
**Unfoldase p97 **	Hexagonal DNA nanocage	Fused to HaloTag	∼10‐fold increase	—	Up to threefold enhancement	—	·Two connected hexagonal nanocages, one houses the p97, the other αCh ·Spatial confinement increases reaction rates ·Physical connection between compartments enhances enzymatic reaction efficiency ·Confinement and unidirectionality reduced off‐target rate by sixfold ·The system can degrade proteins up to 40 kD in size	[20]
**αCh**		Lysine modification using AMAS		—	From 5‐ to 13‐fold enhancement upon higher αCh loading	—	·	
**Amylase**	Triangular DNA origami	Fused to SNAP Tag	Activity of fully assembled system on DNA triangle was up to 30‐fold higher	Slightly lower K_m_	Approximately fourfold increase	Approximately threefold increase	·Best stoichiometry shown for Amylase: Maltase: Glucokinase to be 2: 4: 2, to compensate for the rate‐limiting behavior of maltase ·Maltase showed significant activity enhancement ·Activity differences not driven by pH effects ·Enzymes were also tested at 10 vs. 100 nm apart, and no decrease in the total activity was reported at longer distance, excluding proximity and substrate channeling effects	[64]
**Maltase**				∼16 fold increase in K_m_	∼35‐fold increase	Approximately twofold increase	·	
**Glucokinase**				∼3.4‐fold decrease in K_m_	Approximately threefold increase	∼11‐fold increase	·	
**5 Violacein enzymes **	Linear DNA rod	Covalent enzymes–dCas9 conjugation via SpyTag–SpyCatcher, then the dCas9 binds DNA via guide RNA	∼3.2‐fold increase when all 5 enzymes are on the same DNA rod. ∼1.8 ‐fold increase when all enzymes are bound to a separate DNA rod.	—	—	—	·Binding enzyme to DNA nanostructure plays a role in modulating their activity, regardless of their proximity or orientation. ·Proximity has added value in the activity enhancement	[27]

Abbreviations: 1LS, One‐layer sheet; αCh, Alpha‐chymotrypsin; ADH, Alcohol dehydrogenase; AGO, Amylglucosidase; ß‐Gal, ß‐galactosidase; CA, Carbonic anhydrase; G6PDH, Glucose‐6‐phosphate dehydrogenase; GOx, Glucose oxidase; HRP, Horseradish peroxidase; ICDH, Isocitrate dehydrogenase; LDH, Lactate dehydrogenase; M.TaqI, Methyltransferase; MDH, Malate dehydrogenase; OAD, Oxaloacetate decarboxylase; RuBisCO, Ribulose‐1,5‐bisphosphate carboxylase/oxygenase; XDH, Xylose dehydrogenase; XK, Xylose kinase; XR, Xylose reductase; Sulfo‐SMCC, Sulfosuccinimidyll 4‐(N‐maleimidomethyl)cyclohexan‐1‐carboxylat; SPDP, Succinimidyl 3‐(2‐Pyridyldithio)propionate; BCL‐014, Azide‐N‐hydroxysuccinimide ester; GMBS, N‐[γ‐maleimidobutyryloxy]succinimide ester; sgRNA, Single‐guid RNA; Sulfo‐EMCS, N‐ε‐maleimidocaproyl‐oxysulfosuccinimide ester; AP, P‐aminophenol; P, Phenol; HBA, P‐hydroxybenzoic acid; TMB, Tetramethylbenzidine; oPD, O‐phenylenediamine; ABTS, 2,2′‐azino‐bis(3‐ethylbenzothiazoline‐6‐sulfonic acid); sAAPFpNA, N‐Succinyl‐Ala‐Ala‐Pro‐Phe‐p‐nitroanilide; p‐NPA, P‐nitrophenyl‐acetate; p‐NPB, P‐nitrophenyl‐butyrate; p‐NPV, P‐nitrophenyl‐valerate.

### 3.1 Proximity and Substrate Channeling

**FIGURE 3:**
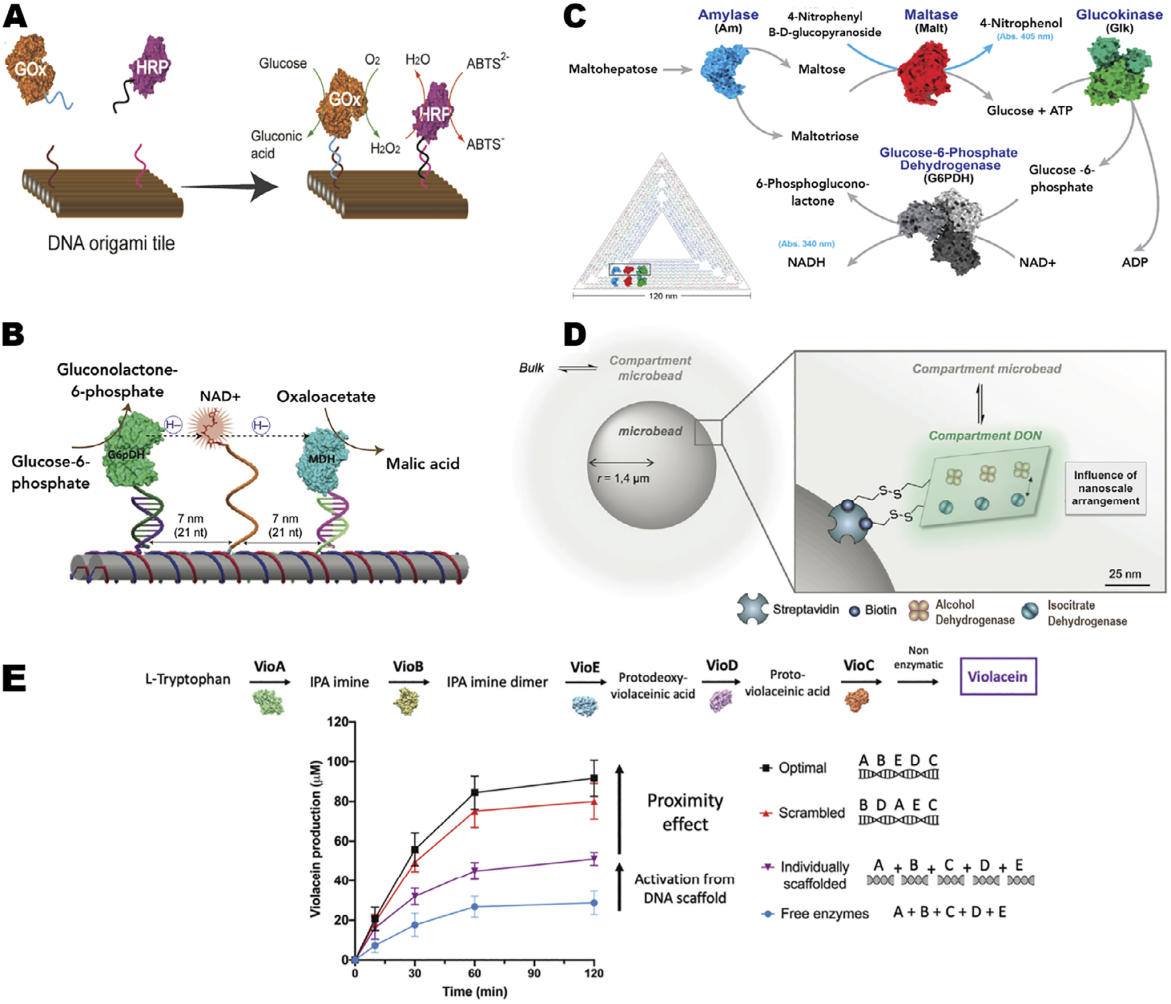
Enhancing enzymatic cascade reactions through DNA nanostructure‐mediated proximity: (A) GOx and HRP co‐localized on rectangular DNA origami achieve optimal activity at 10–20 nm inter‐enzyme spacing. GOx oxidizes glucose to generate H_2_O_2_, fueling HRP‐catalyzed ABTS oxidation [49]. (B) G6PDH and MDH are positioned 7 nm apart with a NAD^+^‐linked DNA strand bridging the enzymes to promote substrate handoff from glucose‐6‐phosphate to malic acid [54]. (C) A triangular DNA origami (∼120 nm per edge) organizes a four‐enzyme cascade (Am, Malt, Glk, and G6PDH), enabling efficient stepwise carbohydrate conversion and substrate channeling [64]. (D) Alcohol and isocitrate dehydrogenases are arranged on DNA scaffolds within microbeads, demonstrating enhanced catalysis via nanoscale spatial control [63]. (E) Sequential conversions from L‐tryptophan to violacein are catalyzed via the violacein biosynthetic pathway (VioA–E). The lower time‐course analysis reveals that optimal scaffolded arrangements significantly improve yield compared to scrambled, individually tethered, or free enzymes (The error bars represent the standard deviation, n ≥ 3) [27]. GOx: glucose oxidase 160 kD, HRP: horseradish peroxidase 44 kD, G6PDH: glucose‐6‐phosphate‐dehydrogenase 100 kD, MDH: malate dehydrogenase 70 kD, Am: amylase 59.8 kD, Malt: maltase 70.9 kD, Glk: glucokinase 37.1 kD, lactate dehydrogenase 106.4 kD, isocitrate dehydrogenase 46.4 kD, VioA‐E: violacein‐producing five enzymes. (A) Reproduced with permission [49]. Copyright 2012, ACS. (B) Reproduced with permission [54]. Copyright 2014, Springer Nature. (C) Reproduced with permission [64]. Copyright 2019, ACS. (D) Adapted with permission [63]. Copyright 2024, Wiley. (E) Adapted with permission [27]. Copyright 2020, RSC.

One of the most widely studied hypotheses on the enhancement of enzymatic activity in or on DNA nanostructures is that of proximity and stabilization of the hydration layer. One of the earliest [28] and most commonly employed enzyme pairs to test this hypothesis has been GOx and HRP. In this cascade reaction, GOx oxidizes D‐glucose with oxygen, producing gluconolactone and hydrogen peroxide (H_2_O_2_). Then HRP utilizes the H_2_O_2_ to catalyze the oxidation of the dye [2,2′‐azino‐bis(3‐ethylbenzothiazoline‐6‐sulfonic acid)] (ABTS) into the radical cation ABTS^.+^, which is spectrophotometrically monitored (Figure 3A). Already in 2009, Willner and coworkers showed that tethering GOx and HRP in close proximity to a DNA tile surface increased their activity [29]. Notably, the observed increase was distance‐dependent, with smaller distances of ∼13 nm between the two enzymes resulting in a stronger activity enhancement compared to larger distances of ∼30 nm [29]. The authors further showed that the observed enhancement was not caused by energy or electron transfer facilitated by the DNA nanostructure [29]. Similar results were also reported by Fu et al. [49, 83], who found an increase in enzymatic reaction if the distance between GOx and HRP was less than 20 nm when arranged on a rectangular DNA origami, while at higher spacing, the distance effect dropped. Investigating the proximity effect further, the authors suggested that the enzyme pair, when in close proximity, has a connected hydration shell layer. This connection locks the diffusion of the reaction intermediate in the vicinity of the enzymes, reducing diffusion to the bulk solution. This so‐called “channeling effect” is assumed to be contributing to the increased apparent catalytic constant [49, 83]. In another study, Gang and co‐workers carried out a systematic study to test different spacings and arrangements between GOx and HRP on a 3D DNA octahedron. They assumed that each strut of the structure is a separate discontinuous origami where ssDNA at the vertices of the octahedron may interfere with substrate channeling [51]. However, as shown by Willner and co‐workers, the DNA scaffold should not interfere directly in substrate channeling [29], but substrate channeling is facilitated by a shared or proximal hydration shell [49, 55]. In their study, Gang and co‐workers showed that higher activity could be observed at 5 nm distance between the enzyme pair compared to 30 nm. Nevertheless, the authors confirmed that tethering enzymes to the DNA nanostructure was the major driver for different catalytic activity, rather than the enzymes’ spacing or arrangement on the octahedrons [51].

Furthermore, the proposed hydration shell effect was considered to play a major role in the alteration of the enzymatic activity of not only enzyme pairs, but also single enzymes in a packed state. For example, both carbonic anhydrase (CA) and xylose reductase (XR) were reported to exhibit accelerated reaction rates when assembled in a packed state with ∼2 nm inter‐enzyme distance on a rectangular DNA origami [39]. Dinh et al. proposed that the entropic force of water confined to the surface of the enzyme molecules increases the local substrate concentration, hence enhancing the reaction. This effect is closely related to the shared hydration shell hypothesis that facilitates “substrate channeling” [39]. However, it was also shown that overcrowding must be avoided to prevent the deactivation of the respective enzymes [84].

Besides proximity and a shared hydration shell, Yan and co‐workers introduced a different way to achieve a substrate channeling effect by employing a flexible DNA arm connected to a required co‐factor, able to “swing” between the two enzymes glucose‐6‐phosphate dehydrogenase (G6PDH) and malate dehydrogenase (MDH) immobilized on a DNA tile [54]. Briefly, G6PDH catalyzes the oxidation of glucose‐6‐phosphate to gluconolactone‐6‐phosphate in the presence of the cofactor nicotinamide adenine dinucleotide (NAD^+^), which gets reduced to NADH. Subsequently, MDH catalyzes the reduction of oxaloacetate to malic acid using the NADH produced by G6PDH (Figure 3B). To control the channeling of the cofactor system NAD^+^/NADH between the enzyme pairs, the authors used a flexible DNA strand attached halfway between G6PDH and MDH [54]. The DNA strand was composed of a poly T20 sequence, modified with NAD^+^. The two enzymes were positioned at different distances (7, 14, and 21 nm from the swinging arms) [54]. Results showed that the shorter the distance, the better the reaction performance, reaching 90‐fold enhancement compared to having the cofactor NAD^+^ freely mobile in the system [54, 62]. Also, when increasing the number of NAD^+^ molecules from one to four, the enzymes exhibited even better activity. This was especially the case for G6pDH, which exhibited a linear increase in its activity (up to fourfold), whilst there was only a twofold increase for MDH catalytic activity when the system incorporated 4 cofactor molecules [54]. Similar results were reported earlier by Willner and co‐workers. In their work, glucose dehydrogenase (GDH) and NAD^+^ were tethered to DNA tiles via DNA strands. While the DNA strand used to tether GDH had a fixed length, NAD^+^ was tethered using different strands with varying lengths. Results showed that when NAD^+^ was tethered via shorter strands, GDH displayed an accelerated catalytic capacity attributed to the enzyme‐cofactor proximity [29].

Despite several studies showing enhanced reaction rates of enzymes placed in close proximity on a DNA nanostructure, other studies to further support the proximity hypothesis were unable to do so, with very incoherent and inconsistent results between different enzyme systems and different origami structures, making it difficult to pinpoint the exact mechanisms of the observed enzymatic activity enhancement. For example, to validate the proximity hypothesis without using DNA nanostructures, Hess and coworkers chemically connected GOx and HRP at a distance of 2 nm using a short oligo strand system [52]. Unexpectedly, their catalytic activity was not enhanced, even though the proximity condition was fulfilled. The researchers therefore proposed that an apparent negative pH shift in the vicinity of a DNA origami surface, rather than the proximity effect, was the cause of the previously observed catalytic enhancement. Accordingly, given the optimal acidic pH of 5.5 for GOx and 6 for HRP, this could be a major contributor to the observed enhanced enzymatic activity on DNA nanostructures [52]. Another study suggesting that proximity may not be the main factor for enhanced enzymatic activity was conducted by Klein et al. In their work, the authors examined the catalytic activity of the cascade reaction between amylase, maltase, and glucokinase attached to a planar triangular DNA origami [64] (Figure 3C). In this triple cascade reaction, amylase hydrolyzes every other alpha‐1,4 glycosidic link in linear starches (maltoheptaose in this study). The reaction starts at the non‐reducing end of the molecule until maltose is formed. The central alpha‐1,4 glycosidic linkage in maltose is then hydrolyzed by maltase to yield two glucose units. Finally, glucokinase phosphorylates glucose to glucose‐6‐phosphate as the first step in glycolysis. The authors tested seven different enzyme configurations with varying distances between the three enzymes. Interestingly, even when the enzymes were positioned as far apart as possible at the vertices of the triangle, there was a significant increase in overall activity, with an approximately 19‐fold increase compared to the free reaction components [64]. This was mainly attributed to the individually increased activity of maltase on the DNA origami, with no dependence on the spacing of the enzymes on the structure [64]. Since both amylase and maltase have similar optimal pH (7 and 6.5, respectively), one would have expected a similar enhancement effect for both enzymes, if indeed the increased negative charge near the origami surface caused the enhancement of activity, as hypothesized in the aforementioned study. However, this was not the case. Therefore, another mechanism for the observed enhancement effect must play a role, which was, however, not further investigated.

Other studies showing that the proposed proximity effect cannot explain the behavior of all enzyme systems include the work of Niemeyer and co‐workers, who observed no significant increase in enzymatic activity after assembling the enzyme pair alcohol dehydrogenase (ADH) and isocitrate dehydrogenase (ICDH) on a DNA origami [63]. ADH stereoselectively catalyzes the reduction of the substrate 5‐nitrononane‐2,8‐dione (NDK) to (R)‐syn/anti‐hydroxyketones(≈60:40). Simultaneously, ICDH oxidizes isocitrate while NADP^+^ is continuously reduced to NADPH, which is essential for the carbonyl reduction of NDK by ADH. The results indicate that the nanoscale proximity of the enzymes on a rectangular DNA origami sheet did not result in a significant increase in catalytic activity. However, when the enzyme‐decorated DNA origami sheets were further conjugated to microbeads, an effect was observed (Figure 3D). In this case, the highest activity was observed when enzymes were co‐assembled on the rectangular DNA nanostructures arranged within the diffusion layer of the microbeads [63]. The authors hypothesized that the intrinsic reactivity of ADH is enhanced by local charge states near the enzyme, potentially coupled with additional conformational stabilization, upon immobilization on the DNA origami nanostructure within the microbead compartment.

In another study, Ke et al. assembled two different cascade reactions on the same rectangular DNA origami: G6PDH combined with MDH, and G6pDH combined with LDH [57]. G6PDH utilizes NAD^+^ in the oxidation of glucose‐6‐phosphate, and the resulting NADH co‐factor could be used by either MDH or LDH in the reduction of oxaloacetate to malic acid or pyruvate to lactate, respectively. Upon testing various distance effects on the two separate coupled reactions (15, 20, and 25 nm), it was found that the G6PDH/MDH cascade had higher activity at a 25 nm inter‐enzyme separation, while the G6PDH/LDH cascade had the highest activity at 20 nm [57]. These results, suggesting that a larger distance resulted in higher enzymatic activity, are once again contradictory to many other works supporting the proximity hypothesis.

Morii and co‐workers also aimed to study the proximity effect by mimicking the effects of proximity in the cyanobacterial carboxysome, the most efficient CO_2_‐fixing organelle in nature [58]. Carboxysomes are the natural reserve of ribulose‐1,5‐bisphosphate carboxylase/oxygenase (RuBisCO) and CA. RuBisCO is responsible for the first step of CO_2_ fixation by incorporating it into the sugar ribulose‐1,5‐bisphosphate (RuBP). Meanwhile, the companion enzyme CA catalyzes the accumulation of concentrated CO_2_ inside the carboxysomes to boost RuBisCO activity and specificity [85]. To mimic the synergy of the RuBisCO/CA enzyme pair synthetically and investigate how their proximity affects activity, Morii and co‐workers assembled the two enzymes on a rectangular DNA origami [58]. Results showed that the proximity of RuBisCO and CA alone was insufficient to boost RuBisCO activity. On the contrary, co‐assembly reduced RuBisCO activity. The authors argued that, potentially, in the natural carboxysome it is not merely proximity but the ratio of CA: RuBisCO, closely confined in a packed state, which affects activity [86]. This study again suggests that proximity alone would not cause the enhancement reported in other studies. Yet other factors like confinement could play a role in modulating the catalytic activity. This will be discussed in a later section. Another study, again by Morii and co‐workers, found further conflicting results. While the authors could show that xylose reductase and xylitol dehydrogenase (XR/XD), involved in the xylose metabolic pathway indeed showed an improved performance when immobilized at close proximity on a rectangular DNA origami [53], a cascade of a xylitol dehydrogenase and a xylulose kinase (XD/XK) arranged on a 3D DNA nanostructure showed almost no correlation between the overall activity and the spatial distance of the immobilized enzymes [61]. To explain the observed contradictory behavior, Morii and co‐workers argued that the imbalanced catalytic constant of the later step (XD/XK) could be the reason behind the absence of any enhanced activity, where the downstream enzyme (XK) is already much more active than its counterpart in the cascade [53, 61].

While most studies discussed until now used a two‐enzyme system to study the effect of proximity on enzymatic activity, Clark and co‐workers expanded their work to study an even larger enzymatic cascade reaction. The authors assembled five tandem enzymes of the violacein biosynthesis pathway (Vio A‐E), arranged on a 295‐bp linear dsDNA helix, with 10 nm distance between each enzyme [27] (Figure 3E). Notably, instead of strictly studying the effect of different distances between the individual enzymes, the authors mainly focused on their arrangement concerning the “correct” sequence in the cascade reaction. The conversion of L‐tryptophan to the purple‐colored violacein pigment was quantified in three different positioning variations on the dsDNA, and each compared to the freely floating enzymes in solution: a) each single enzyme of the cascade was tethered to a separate DNA helix, b) the five enzymes were arranged on the same DNA helix in the correct order of the catalytic sequence in an “optimal” arrangement, and finally c) the five enzymes were placed in a “scrambled” arrangement on the same DNA helix. In the first case, the authors reported a 1.8‐fold increase in overall activity of the cascade, which was attributed to the simple tethering of each enzyme to a separate dsDNA. As for the second and third cases, there was a 3.2‐fold increase in the final violacein pigment production, with an insignificant difference between the optimal and scrambled arrangement. The authors concluded that the close proximity of the enzymes, together with their conjugation to DNA, contributed to the overall higher activity of the metabolon. However, to confirm the concrete underlying mechanism, more in‐depth studies would be needed [27].

Furthermore, controlling the distances between hosted enzymes within DNA nanostructures has also been closely connected to the geometry of the DNA nanostructure itself. Various DNA geometries and shapes have been designed to study how the manipulation of the physical arrangement at the nanoscale changes the catalytic behavior of enzymes of interest and affects the physicochemical performance of the whole system. For example, a study observing the three‐enzyme cascade consisting of MDH, oxaloacetate decarboxylase (OAD), and LDH, found that a three‐point star‐patterned arrangement significantly influenced the efficiency of the cascade compared to the linear arrangement [55]. Briefly, MDH catalyzes the oxidation of malic acid to oxaloacetate in the presence of the cofactor NAD^+^, which gets reduced to NADH. Subsequently, OAD breaks down the oxaloacetate into pyruvate, preventing a backward reaction and the reformation of malic acid. Finally, LDH utilizes the pyruvate in the presence of NADH to produce lactic acid. The first step of this catalytic cascade is highly unfavored compared to the backward reaction, where MDH catalyzes the reduction of oxaloacetate into malic acid with a much faster reaction rate constant (∼16‐fold difference in turnover number). The three enzymes were positioned each on the tip of a three‐point star DNA nanostructure, with an average inter‐enzyme distance of ∼12 nm [55]. This resulted in a fivefold increase in the end product lactate. When compared to linear sequential enzyme arrangement at different distances (7, 14, 21, and 28 nm), arranging the enzymes in a three‐point star pattern proved to be superior in the case of this triple‐coupled enzyme cascade. This particular alignment plays in favor of the forward reaction of MDH catalyzing the oxidation of malic acid to oxaloacetate while reducing NAD^+^ to NADH, which normally holds a positive Gibbs free energy, making it readily reversible [55]. The coupled proximity (achieved by maintaining equidistance from each pair of the trio) guarantees that intermediates of the rate‐limiting step are continuously depleted, enabling the forward reaction to proceed faster [55].

Likewise, variation of the spatial arrangement of tandem enzymes in a 3D configuration on a tetrahedral DNA framework boosted the cascade catalytic reaction compared to 2D DNA structures, for the two‐enzyme system (GOx/HRP) or the three‐enzyme system (amylglucosidase (AGO)/GOx/HRP) [56]. The 3D assembly allowed the authors to deploy various molar stoichiometries for the combination of the three enzymes, to allow for the compensation for the catalytically limiting step [56].

Taken all together, the proximity of enzymes in a cascade, ensuring a connected hydration shell locking substrates and intermediates, could only be applied to certain enzymes coupled to different DNA nanostructures. However, the proximity hypothesis falls short in generalization, as many systems did not perform as expected even when the proximity condition was fulfilled. In the following sections, we will discuss other factors thought to contribute to the modulation of enzymatic activity coupled to DNA nanostructures.

### 3.2 Surface Characteristics (pH and Substrate Affinity)

It has been suggested previously in multiple works that the highly negative charge of the DNA phosphate backbone, especially on the densely packed surface of a DNA nanostructure, is contributing to the modulation of the catalytic activity of immobilized enzymes. This effect has been hypothesized to take place via two potential routes: (i) a direct influence on the enzyme by providing a confined and modified microenvironment pH on the highly polyanionic DNA. Water molecules near the DNA surface create a tight, 0.3 nm‐thick hydration layer of hydrogen bonds, resulting in an apparent decreased pH close to the DNA surface [87], which is different from the bulk solution. This effect is expected to work in favor of enzymes having acidic pH‐dependent activity (pH 6 or lower). Generally, the hydration shell surrounding a protein plays a central role in governing molecular recognition, folding dynamics, and proton‐transfer processes [88]. For DNA, two layers of hydrogen bonds have been proven to form at 0.3 and 0.8 nm away from the DNA backbone. Beyond that, water molecules gradually lose their structure and transition to bulk behavior, while experiencing some effect from the strong electrostatic field created by the DNA [87]. In this context, most enzymes bound to DNA nanostructures, at 5 or 6 nm distance from the DNA surface, would be positioned beyond the range of the structured hydration layer. In all cases, one could hypothesize that the highly compact order of double helices in DNA origami nanostructures may extend the influence of the electrostatic field to a greater distance into the bulk solution. So far, we know that the hydration layer surrounding the DNA changes when DNA changes conformation (e.g. from a relatively “unpacked” state to a more compactly folded one) [89, 90, 91]. However, the literature on this topic is still somewhat sparse—especially when it comes to comparing “ordinary” duplex DNA in solution vs highly ordered/condensed DNA, such as that in DNA nanostructures. Most hydration studies focused on simple duplex DNA—either in solution, or as crystals/films. Meanwhile, for DNA nanostructures, the research tends to focus on macro‐properties: stability under salt / chemical treatment, aggregation, binding, folding yield, mechanical integrity—not explicitly on hydration shell dynamics. Therefore, further research into this topic is required to allow for a proper explanation of the influence of the hydration shell around DNA nanostructures on enzymatic activity.

On the other hand, route (ii): an indirect influence by creating a locally substrate‐enriched microenvironment via e.g. electrostatic interactions between the DNA and the substrate. Substrate enrichment increases enzymatic activity by raising the effective local substrate concentration near the enzyme, which increases encounter frequency, lowers escape probability, and shifts kinetic parameters to favor faster turnover rates even if the intrinsic affinity is unchanged or slightly reduced.

#### 3.2.1 Multi‐Enzyme Systems

**FIGURE 4:**
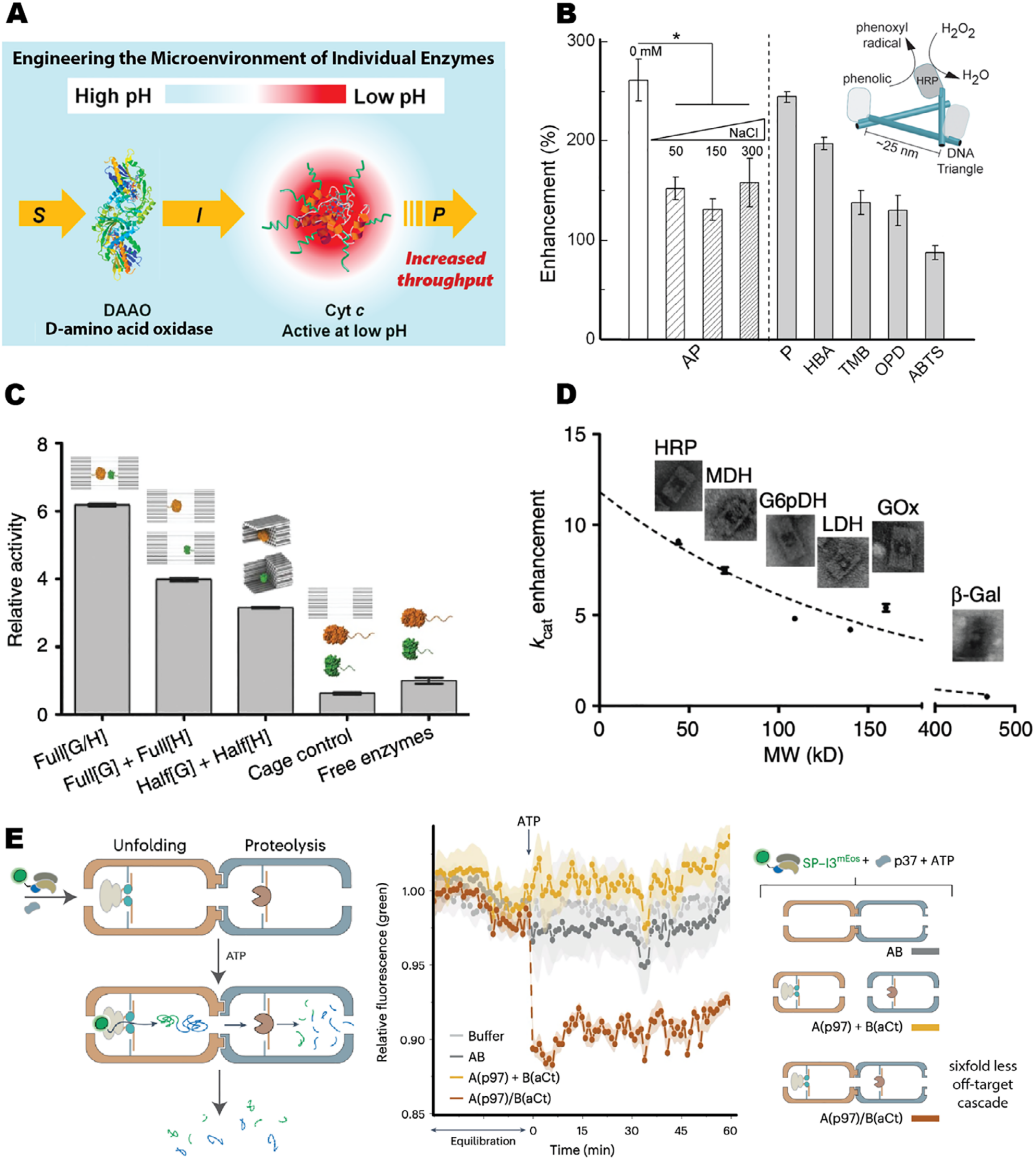
Mechanisms contributing to enhanced enzyme activity on DNA nanostructures: (A) Local pH modulation enables compartmentalized cascade reactions by creating a low‐pH microenvironment around Cyt c, while DAAO remains active at higher pH [26]. (B) HRP activity is enhanced on DNA triangles in a substrate‐ and salt‐dependent manner, with optimal catalysis at moderate substrate–DNA interactions and low salt concentrations (The error bars represent the standard deviation, *n* ≥ 3, * *p* ≤ 0.05) [97], AP: p‐aminophenol, P: phenol, HBA: p‐hydroxybenzoic acid, TMB: tetramethylbenzidine, OPD: o‐phenylenediamine, ABTS: 2,2′‐azino‐bis(3‐ethylbenzothiazoline‐6‐sulfonic acid). (C) DNA cages improve cascade efficiency of GOx/HRP through spatial confinement and enzyme co‐localization (The error bars represent the standard deviation, *n* ≥ 3) [47]. (D) Inverse correlation between enzyme size and catalytic enhancement on DNA cages: smaller enzymes display better catalytic turnover rate (k_cat_) enhancement (The error bars represent the standard deviation, *n* ≥ 3) [47]. (E) A modular DNA nanomachine controls ATP‐dependent protein unfolding and proteolysis. Full cascade assembly (p97/aCt) enables efficient target degradation with minimal off‐target activity [20]. DAAO: d‐amino acids oxidase 74 kD, Cyt C: cytochrome C 12 kD, GOx: glucose oxidase 160 kD, HRP: horseradish peroxidase 44 kD, MDH: malate dehydrogenase 70 kDa, G6PDH: glucose‐6‐phosphate‐dehydrogenase 100 kDa, LDH: lactate dehydrogenase 140 kDa, ß‐Gal: ß‐galactosidase 450 kDa, p97: ATPase p97‐valosin‐containing protein 582 kD, aCt: alpha.chymotrypsin 25 kD. (A) Reproduced with permission [26]. Copyright 2017, ACS. (B) Reproduced with permission [97]. Copyright 2013, ACS. (C, D) Reproduced with permission [47]. Copyright 2016, Springer Nature. (E) Adapted with permission [20]. Copyright 2024, Springer Nature.

Route (i) was initially investigated experimentally by Hess and co‐workers using polymethacrylic acid (PMMA), which carries a negative charge similar to DNA [26]. They demonstrated that conjugating Cytochrome C (Cyt C, optimal activity pH 4.5) to the negatively charged PMMA allowed it to sustain high activity over a wider bulk solution pH ranging from 4 to 7 (Figure 4A). This was attributed to the abundance of carboxylic groups carried by the PMMA in the direct vicinity of Cyt C, which created an artificial apparent lower pH at the active site. This in turn allowed the modified Cyt C to function in harmony with D‐amino acid oxidase (DAAO), another enzyme requiring higher pH for catalysis. The modification of Cyt C with PMMA enabled a 10‐fold increase in the cascade throughput at pH 8^26^.

Testing the pH hypothesis on DNA nanostructures, Lin et al. studied XR and XD arranged on a DNA origami, having optimal pHs at around 6 and 8, respectively [60]. Their results indicated that the pH near the DNA surface is 0.8 points lower than in the bulk solution, or 0.5 points lower at 6.7 nm away from the DNA surface [60]. Taking this into account, a higher enzymatic activity would be expected for XR with an optimum pH of 6. However, both enzymes, despite their different optimal pHs, showed almost a fourfold increase in their turnover rate, indicating that the presumed lower microenvironment pH could not be the major driving factor for enhanced activity in this case [60]. Similarly, upon placement of amylase, maltase, and glucokinase on a planar triangle DNA nanostructure, mainly maltase displayed significantly enhanced activity, despite amylase and maltase having similar optimal activity in the pH range of 6.5–7 [64]. In another study by Kröll et al., ketoreductase Gre2p and ADH were tested on a planar 1LS DNA coupled to microbeads, with optimal literature pH 5 and 7–8, respectively [92, 93, 94] (although in the discussed study, both enzymes showed similar pH‐activity profiles). Astonishingly, Gre2p exhibited 20% lower activity, and ADH showed 40% higher activity upon conjugation to the DNA origami, despite the higher optimum pH hypothesis [74]. In summary, thus far there have been no conclusive studies confirming this hypothesis with respect to DNA nanostructure‐tethered enzymes. In contrast to systems in which pH effects can be decoupled and interrogated in isolation—such as the modulation of Cyt C using PMMA [26]—the DNA nanostructures present a multifaceted microenvironment. Here, factors including hydration layers, substrate enrichment, nanoscale confinement, and structural stabilization act in concert, rendering pH one contributing parameter within a complex, interdependent network rather than a singular, all‐or‐none determinant of enzymatic behavior.

#### 3.2.2 Single‐Enzyme Systems

At the same time, route (ii), assuming DNA‐substrate interactions, has also been the subject of intense investigations: different substrates are known to bind to DNA through the major and minor grooves, through electrostatic interactions with the phosphate backbone, or through intercalation between DNA base pairs [95]. Making use of this, Wheeldon and coworkers conjugated HRP to DNA tiles and assessed its activity using three different substrates: p‐aminophenol (AP), tetramethylbenzidine (TMB), and ABTS. The authors found that micromolar binding interactions between the substrates and the DNA led to their increased local concentration in the proximity of the HRP active site and thus increased the apparent catalytic performance of the enzyme [25, 96]. Similarly, the authors further tested the effect of different distinctly charged substrates (AP and TMB (+1), o‐phenylenediamine (oPD) (+2), p‐hydroxybenzoic acid, and ABTS (−1)) on HRP activity when bound to triangular DNA nanostructures (Figure 4B). The study found that the performance of HRP exhibited a Sabatier‐like trend, with enhanced catalysis recorded at moderate substrate−DNA binding energy, which decreased when the interaction was too strong or too weak [97]. A similar study was conducted recently by Kostiainen and co‐workers on HRP confined in a DNA cage, coated with positively charged viral capsid proteins (VCP) [37]. In their study, oPD (+2), TMB (+1), and ABTS (−1) were used as substrates in the investigation of the catalytic performance when HRP was confined in DNA cages with varying charges and porosity depending on the coating thickness and composition of the VCPs [37]. The affinity of the substrates to HRP (represented by the K_m_ constant) as well as the apparent catalytic activity and V_max_ changed with different DNA origami coatings with positively charged VCPs. Although there was no clear pattern, the study highlights the possibility to modulate enzymatic activity by carefully designing the carrier system [37].

Further, when the esterase activity of CA (in a packed state) tethered to rectangular DNA origami was tested using substrates with increasing hydrophobicity of p‐nitrophenyl compounds (‐acetate, ‐butyrate, and ‐valerate), it was shown that the most hydrophobic valerate compound resulted in the highest enzymatic activity. The authors attributed the observed enhanced activity to substrate enrichment, which is strengthened for more hydrophobic substrates at the DNA/enzyme–water interface. Morii and co‐workers argued that hydrophobic substrates exclude more water volume at the interface, which increases entropy, making the enzyme more active with an increased hydrophobicity of the substrate [39]. These results, however, are contradictory to a recent study of the same group, where the authors showed that XR and CA, when tethered on a DNA origami, displayed accelerated catalytic rates with hydrophilic substrates and decelerated rates with hydrophobic substrates. In this study, the substrate's affinity was determined by hydration free energy—rather than the conventional logP values—calculated via a statistical mechanics model. The authors proposed the ordered hydration shell layer near the highly negative DNA surface to be the reason for enriching the hydrophilic substrates near the enzymes tethered to the DNA origami [40]. Earlier in 2016, Yan and co‐workers also proposed that the ordered hydration shell layer at the DNA origami surface plays a role in the apparent enhanced catalytic activity for DNA‐enzyme hybrid nanostructures via increased stability of the enzyme's active conformation [87]. The authors hypothesized that the ordered hydration layers maintain the active conformation state of the enzyme, due to an increased energetic penalty upon unfolding, and this is irrespective of the effect of substrate enrichment [47].

Furthermore, Kosinski et al. explored how the catalytic cycle of thrombin, a serine protease, is influenced by two structural factors: the C‐terminal residue of the substrate and the presence of a DNA nanostructure [41]. The C‐terminal residue affects thrombin allosterically, while DNA nanostructures primarily modulate the enzyme's kinetic behavior through electrostatic interactions [41]. It was found that these interactions can significantly alter the enzyme–substrate interface, often increasing the steady‐state reaction rate in the presence of DNA. The extent of this enhancement depends on the substrate's charge and concentration, with neutral or negatively charged substrates exhibiting faster cleavage due to transition state stabilization and positively charged substrates experiencing destabilization of the enzyme–substrate complex [41]. The presence of DNA affects both binding and activation energies, potentially creating alternative kinetic pathways through ion‐dependent allosteric regulation [41].

These combined results confirm the general influence of charge density over the enzyme active site; nevertheless, the pH of the microenvironment is unlikely to be the root cause of behavioral change in activity recorded for tethered enzymes on DNA nanostructure surfaces, at least it is not the sole mechanism.

### 3.3 Confinement in 3D DNA Nanostructures

As already mentioned previously, spatial confinement can also play a pivotal role in modulating enzymatic activity, mimicking the often crowded and compartmentalized environments found in natural biological systems. Confinement has been employed in synthetic biotechnology for efficiency maximization through the immobilization of enzymes within different materials such as polymers, including DNA, proteins, inorganic nano‐ and microparticles [98, 99, 100]. The primary effect of confinement enhances all mechanisms described earlier. This includes the enrichment of local substrate concentration, which increases the likelihood of enzyme‐substrate interactions and can lead to improved reaction rates, particularly in multi‐enzyme cascade systems. Further, 3D confinement is expected to amplify local proton accumulation in the immediate vicinity of encapsulated enzymes, as the surrounding architecture promotes enrichment from multiple spatial directions. Additionally, confinement can stabilize enzymes structurally by limiting conformational freedom, thus preserving catalytic efficiency over time. DNA nanostructures present an interesting material to study the effect of confinement on enzymatic activity, due to their controllably designable shape and size, as well as complete and nm‐precise addressability. Fu et al. used short DNA nanotubes as well as planar rectangular DNA origami sheets to immobilize only HRP or the GOx/HRP cascade system to study the effect of confinement [50]. For both the single enzyme as well as the two‐enzyme cascade system, higher activity was observed upon confinement in the DNA nanotube compared to the planar DNA origami sheet [50]. The authors proposed that confinement blocks the diffusion of the reaction intermediate to the bulk solution and thus results in a higher local substrate concentration. However, due to the small size of the substrate H_2_O_2_, and the comparably large pores found in DNA origami, this is not likely. Further, Yan and co‐workers tested GOx/HRP in a single nanocage versus single GOx or HRP enzymes in separate nanocages [47]. It was calculated that approximately 65% of enhanced activity was driven by confinement rather than just proximity [47]. The researchers attributed the enhanced activity to the ordered hydration layer near the DNA surface, which contributes to the stabilization of the folded protein and their hydrophobic interactions, as unfolding becomes less favorable [47] (Figure 4C). In another work, a confined and compartmentalized cascade system was developed using a modular nanoreactor composed of two homogeneous building blocks, each encapsulating one enzyme (either GOx or HRP), then assembled at a later stage [23]. Results showed that each monomer, as well as the dimer, maintained catalytic activity with higher activity compared to the control, which in this case was mixing the enzymes and the DNA origami without handles for attachment to keep the enzymes freely floating, suggesting that confinement enhanced enzymatic activity of both the single and the coupled enzyme system [23].

It was further found that, in the case of confinement within a DNA origami nanostructure, the amount and arrangement of helices around an enzyme affected their affinity. As such, a study reported that increased amounts of helix densities, represented by structures made up of the lowest density (a) honeycomb lattice to medium density (b) single‐layer square lattice to highest density (c) double‐layer square lattice in DNA nanocages, correlated with increased activity of the enzyme Glucose‐6‐phosphate dehydrogenase (G6PDH) [47]. The reported enhancement was mainly manifested as a higher turnover rate; meanwhile, substrate affinity did not increase significantly. This suggests that the catalytic core of the G6PDH was directly affected by the DNA confinement, regardless of any substrate local enrichment [47]. Besides the DNA helix packing density, another factor that has been reported to influence enzymatic activity and how strongly an enzyme is influenced by the DNA origami confinement is the actual enzyme size. For this, Yan and co‐workers studied six different enzymes of increasing molecular weights: HRP (44 kDa), MDH (70 kDa), G6PDH (100 kDa), LDH (140 kDa), GOx (160 kDa) and ß‐galactosidase (ß‐Gal, 450 kDa) confined in a DNA origami cage [47]. An inverse correlation was reported between the enzymes’ molecular weight and their activity enhancement, with the highest turnover rate observed for HRP and MDH. Contrastingly, the bulky ß‐Gal exhibited decreased activity upon encapsulation in the nanocage [47] (Figure 4D). To further investigate the apparent deceleration of ß‐Gal upon encapsulation in DNA nanocages, the researchers analyzed the effect of polyphosphates on ß‐Gal and noted that decreased activity for ß‐Gal was reported in a polyphosphate‐rich environment. The authors thus assumed the poor performance of ß‐Gal activity to be due to the negatively charged phosphate backbone in the DNA nanostructure [47].

Interestingly, several studies also found that confinement did not enhance enzymatic activity. E.g., a coupled reaction of XD and XK confined in a 3D DNA origami capable of switching from an open to a closed state did not show any statistical difference in the catalytic activity between the two states, suggesting that confinement in this case did not enhance the activity [42, 61]. Similarly, the placement of the protease alpha chymotrypsin (aCt) in a fully closed DNA reconfigurable nanovault showed decreased activity compared to an open vault [38]. However, when aCt was confined in a hollow hexagonal DNA prism, a 10‐fold enhanced efficiency of the proteolytic digestion could be observed [20] (Figure 4E). Taken together, just as for the other hypotheses, the confinement effect alone cannot be responsible for the often‐observed enhanced activity of enzymes in or on DNA nanostructures and certainly does not hold true for all enzyme systems.

### 3.4 Protection and Stabilization

As a final hypothesis that has been postulated to describe the change in activity observed in DNA‐tethered enzymes compared to freely floating components, it has been proposed that DNA as a support exerts a protective role that shields enzymes from detrimental external factors. Or, DNA could have a stabilizing effect, influencing the active site in the course of catalysis. It is noteworthy to highlight that, compared to other hypotheses reported, a stabilizing effect is very challenging to experimentally test. Lin et al. suggested that the enhanced apparent catalytic activity of scaffolded enzymes was attributed to the prevention of enzymes from adsorbing onto a surface during measurements. In this regard, adding bovine serum albumin (BSA) to the reaction medium as an adsorption scavenger helped retain enzymatic activity for longer periods [60]. However, preventing adsorption cannot explain the great enhancement of enzymatic activity observed on DNA origami structures. As an alternative, the authors suggested a stabilization effect of the enzyme configuration attributed to the ordered hydration layer at the DNA surface [60]. Another important stabilization effect is the protection against enzymatic digestion. In the previously discussed study by Yan and coworkers47, encapsulation within DNA nanostructures was shown to effectively protect enzymes from proteolytic degradation. Specifically, the encapsulated GOx/HRP cascade retained its catalytic activity in the presence of trypsin, with enzymatic activity remaining essentially unchanged before and after protease treatment. This indicates that the DNA nanocage acts as a physical barrier, sterically hindering access of trypsin to proteolytically susceptible sites on the enzymes. In contrast, the free enzyme system exhibited a pronounced reduction in activity—approximately a 50% decrease—following trypsin exposure. Further, in another study investigating the catalytic performance of a tandem reaction between amylase, maltase, and glucokinase attached to a triangular DNA origami, it was reported that maltase's activity was in particular drastically deteriorating when the enzyme was subjected to moderate temperatures of 37°C. However, attachment to the DNA seemed to stabilize the maltase enzyme against this temperature [64]. In addition, it was shown by Kröll et al. that ADH conjugated to a rectangular DNA origami displayed a 40% higher activity, potentially due to the negatively charged DNA attracting protons to the surface, stabilizing the ADH hydrophobic binding pocket. This hypothesis is controversial, however, as ADH has a preferential basic pH for its maximum activity [74, 93]. All in all, it can be concluded that DNA can exhibit some protective properties, but again, this cannot in most cases explain the observed enhanced activity of many enzymes conjugated to a DNA nanostructure.

## 4 Discussion

All above‐reported experiments and observations highlight the great contradiction existing in explaining the observed behavior of DNA‐enzyme hybrid nanostructures. Moreover, attempts to reproduce early results are not always consistent, even when applying the same enzymes, or enzymes that share apparent similar physicochemical properties such as molecular weight, isoelectric points, mechanism of action, or preferential catalytic pH. The collective findings discussed herein challenge the early assumption that spatial proximity or controlled distances enhance enzyme performance for DNA‐enzyme hybrid nanostructures. Theoretically, this has also been supported by McCammon and co‐workers, who showed via molecular simulations that close proximity between enzymes does not necessarily result in optimal reaction rates [101]. Effects of local microenvironment pH and ionic strength have similarly been hypothesized to lead to the observed modulated enzyme/substrate affinity or enhanced catalytic performance via altering the pKa of participating ionizable residues in the reaction of interest. Nevertheless, reported results so far were not consistent across studies for DNA‐enzyme hybrid nanostructures. For DNA‐scaffolded enzymes, the underlying mechanisms can be considerably more complex and also strongly depend on the chosen cascade and specific kinetic properties of the enzymes. In addition, other factors discussed earlier contribute differently to the modulation of activity, such as the ordered hydration layer, substrate enrichment near the DNA surface, and reduced adsorption on reaction vessels.

It has been suggested repeatedly in the literature that a pair of enzymes in proximity share connected hydration shells that lock the diffusion of the reaction intermediate in the vicinity of the enzymes, without diffusing to the bulk solution. This “substrate channeling effect” is assumed to be contributing to the increased apparent catalytic constant. Nevertheless, given the wide use of the GOx/HRP pair as a model for studying the catalytic mechanism upon attachment to DNA nanostructures, it is highly debatable that this particular enzyme pair is the right model to study such effects, because under relevant experimental conditions, diffusion is much faster compared to catalytic reaction rates [102]. First of all, the substrate and intermediate of the GOx/HRP coupled reaction presumably do not suffer from limited diffusion, since the diffusion coefficient of H_2_O_2_ is ∼1430 µm^2^/s [103]. Second, all experiments were carried out at saturation concentrations, exhibiting a substrate‐rich zone of a few micrometers in diameter, centering the enzymes of interest [49, 104], which further excludes substrate diffusion limits. This, however, does not per se rule out the substrate channeling effect as a key mediator in enhancing cascade reactions. Nevertheless, it should be considered only when it can be practically applied: (1) for substrates with limited diffusion, which is unlikely for most small chemical substrates and intermediates, (2) for enzymes suffering from low catalytic reaction rates, such as β‐galactosidase and RuBisCO. Generally, in silico modeling suggests that substrate channeling between two sequential enzymes becomes ineffective when they are separated by more than 2 nm, with less than 25% probability of successful substrate channeling. However, the effective range for substrate channeling can be extended up to 10 nm when electrostatic interactions between the cascade architecture and reaction intermediates facilitate directed diffusion between active sites [101, 102].

Another hypothesis proposes that the ordered hydration shell in the direct vicinity of DNA helices is considered to play a major role in the alteration of the enzymatic activity of single enzymes, especially in a packed state. So far, this hypothesis, although plausible, has also been controversial. The main method applied to presumably disrupt the ordered hydration layer at the DNA surface relies on the addition of large amounts of salts like NaCl, which often also negatively affects the activity of unbound, free enzymes [40, 47]. In such cases, where experimentally the control group (freely floating enzymes) shows similar behavior to the test group (DNA origami‐bound enzymes), a conclusion regarding the contribution of the ordered hydration layer cannot be confirmed. Furthermore, there have been opposing opinions on the role played by the ordered hydration layer near the surface of DNA. Yan and co‐workers hypothesized that the hydration layer contributes to the stabilization of the folded protein and their hydrophobic interactions, as unfolding becomes less favorable, without contributing to the substrate enrichment near the surface [47]. On the other hand, Morii and co‐workers attribute the behavioral change to the interplay between the layer and the degree of hydrophilicity of the substrate, which affects its local up‐concentration near the vicinity of the enzyme [40]. Either or both explanations could be true, but so far have not been substantially validated by different groups.

On a different note, other factors at play are related to the DNA nanostructure design and sequences of handles used to anchor the enzymes of interest. The disparity in DNA nanostructures used as different models across individual experiments is assumed to play a major role in the discrepancy in reported findings. For example, the employment of planar rectangular or triangular DNA nanostructures as a pegboard to tether enzymes could be of interest owing to their simple design and high reproducibility. Nevertheless, assembling enzymes with disproportionally large volumes and molecular weight on a DNA rectangular sheet might not be a proper representation of the influence of DNA on the enzymes of interest. DNA nanostructures in a planar form provide a rigid and geometrically defined platform for spatially organizing biomolecules. While this can be advantageous for studying nanoscale positioning and proximity effects, the physical mismatch between the scaffold and the enzyme's size and shape may lead to artifacts or misinterpretation of results. Enzymes with complex tertiary and quaternary structures often exhibit anisotropic shapes, dynamic surface conformations, and large hydrodynamic radii that may extend well beyond the plane of the DNA sheet. This spatial discordance could result in limited or nonuniform interaction with the DNA surface, reducing the intended confinement effects or even introducing steric clashes and conformational constraints. Moreover, attaching such large enzymes at fixed positions on a relatively small and flat surface might neglect critical aspects of native‐like environments, such as the presence of three‐dimensional confinement, dynamic crowding, and orientation freedom. On this note, various large enzymes in DNA nanostructures were reported: (1) Form II RuBisCO (∼115 KDa) on a rectangular DNA origami [58], (2) GOx (160 KDa) on rectangular DNA origami, nanorods, triangles, nanocages, and others [23, 47, 49, 50, 56], (3) β‐galactosidase (450 KDa) in DNA nanocapsules [47] and DNA branches [24], (4) Halo‐tagged‐p97 (∼780 KDa) in hollow hexameric DNA nanocages [20]. As discussed previously, Yan and coworkers [47] reported an inverse correlation between an enzyme's molecular weight and its activity enhancement upon encapsulation in DNA nanocages. Nevertheless, when comparing results from cross‐studies, both RuBisCO [58] and β‐gal [47] exhibited decreased activity, although p97 showed a ∼10‐fold increase in activity, despite its much larger size, contradicting the observed size‐correlation reported by Yan and co‐workers [20]. This discrepancy can likely be attributed to differences in the underlying DNA origami design employed in the respective studies. β‐gal (∼ 18 × 14 × 9) nm^3^ [105] was investigated within a relatively compact nanocage featuring an internal cavity of approximately (∼ 20 × 20 × 20) nm^3^ [47]. When accounting for ssDNA conjugation via lysine modification, the effective hydrodynamic dimensions of β‐gal are expected to increase further, thereby imposing additional steric constraints. Such spatial limitation is likely to restrict the intrinsic conformational flexibility of the enzyme during catalysis, potentially limiting its activity. In contrast, the ATPase p97, with an approximate diameter of ∼16 nm and an additional ∼12 nm contribution from the DNA handle, was encapsulated within a substantially larger DNA nanocage with an internal diameter of at least 30 nm [20]. This expanded cavity provides sufficient spatial allowance to preserve the dynamic motions of the enzyme and facilitates unhindered substrate access to the active site. Collectively, these observations highlight the critical role of nanostructure geometry and confinement volume in modulating enzymatic function within DNA nanostructures. It is therefore essential to conduct carefully designed further studies to harmonize all these observations.

Moreover, the experimental setup can often affect the data collection and interpretation of results. For example, FRET pairs are often used as a method to validate the position of the enzymes as predesigned, by attaching the fluorophores to the same DNA sequences as those used to tether the enzymes of interest. Without taking into consideration the major difference in sizes between the enzyme molecules and fluorophores, this could interfere with the accurate interpretation of assumed results [51]. Even when using gold NPs as a model to study uptake, the results could not be cross‐applied to enzymes of similar size to the NPs used, which leads to an approximation of the number of enzyme molecules per each DNA nanostructure in some studies [37]. Also, the variation in chemical and/or molecular modification applied to enzymes to allow for tethering to the DNA nanostructure contributes to the discrepancy of reported results in terms of enzyme kinetics, turnover rates, and substrate affinity. In one study, HRP was incorporated into a DNA cage with 20%–30% encapsulation efficiency [35]. Meanwhile, fluorescently labeled HRP failed to be encapsulated when applied in the same system, demonstrating the critical effect of the conjugated fluorophore [35]. In another study, using the superfolder green fluorescent protein (sfGFP), Abdallah et al. showed that covalently fusing this negatively charged fluorescent protein to ADH influenced the ionic strength in the vicinity of the enzyme's active site, resulting in enhanced NAD(P)^+^ cofactor binding and stabilizing the transition state [106]. Therefore, it is essential to also consider the nature of the fluorophore, which can greatly affect the enzymatic behavior and interaction with the DNA nanostructure. This reflects again that reported methods are not necessarily transferable to different enzymes with dissimilar properties, and also incorporation of a molecule of interest into nucleic acid nanostructures is governed by a plethora of physicochemical factors beyond the sheer size or molecular weight. Given that nonspecific chemical modification (e.g., modification of Lys or Cys residues) of enzymes is often used for DNA conjugation, there is no control over the final orientation of the active site when tethered to the origami, which contributes to the nuances when interpreting the mechanism of activity. An additional experimental constraint arises from the buffer conditions required to preserve the structural integrity of DNA nanostructures, which may not coincide with the optimal operating conditions of the encapsulated enzymes. Typically, DNA origami constructs are maintained in Tris‐ or phosphate‐based buffers within a mildly alkaline pH range (between 7 and 9), supplemented with millimolar concentrations of Mg^2^^+^ to prevent structural collapse or aggregation. As summarized in Table 1, the majority of enzymes investigated to date remain functionally compatible with these conditions, enabling systematic studies without compromising DNA origami stability. However, this compatibility often comes at the expense of operating under suboptimal enzymatic conditions. For instance, enzymes with acidic pH optima—such as GOx (optimal pH ∼5.5) and HRP (optimal pH ∼6)—are routinely assayed within the standard DNA origami buffer to maintain nanostructure integrity. Consequently, in GOx/HRP cascade systems, experimental conditions are typically dictated by the structural requirements of the DNA nanostructure rather than by the intrinsic catalytic optima of the enzymes. This trade‐off underscores a fundamental limitation in the current DNA‐based platforms, where maintaining structural fidelity may constrain the ability to fully realize maximal enzymatic performance.

Another challenge for DNA origami structures is their inherent porosity due to their mesh‐like lattice arrangement of the DNA helices. This structural porosity allows small molecules to diffuse freely through interhelical gaps, typically spaced 1–2 nm apart. While such permeability facilitates the exchange of reagents and substrates in enzymatic applications, it becomes a limitation when selective encapsulation or molecular gating is required. In a recent study, Scherf et al. explored the ability of DNA origami nanotubes sealed with DNA lids to encapsulate freely diffusing protein cargo such as streptavidin and BSA [107]. Despite the geometric closure of the nanocages, they found that medium‐sized proteins (∼5–8 nm) escaped rapidly, with poor retention observed over time [107]. Coarse‐grained simulations confirmed that the escape occurred primarily through dynamic gaps at imperfectly sealed interfaces, particularly at lid–tube junctions where repulsive DNA overhangs permitted transient openings [107]. Re‐designing the lids to eliminate consecutive repulsive ends significantly reduced leakiness, demonstrating that DNA origami porosity is not just intrinsic but also tunable through interface engineering. These findings underscore the need for careful structural refinement when using DNA nanostructures for cargo encapsulation, especially when the goal is long‐term retention or controlled release [107]. Further strategies that may reduce DNA origami leakiness, such as coating with silica or other proteins [37] or introducing additional crossovers, may be beneficial for further studies [107].

Another overlooked aspect is the diffusivity of enzymes during catalysis. Enzymes typically function through random Brownian diffusion in solution until they encounter their substrate, with passive diffusion constants for proteins typically between 10 and 100 µm^2^/s. On the other hand, some enzymes show catalysis‐enhanced diffusion or self‐propulsion where they transiently increase their effective diffusivity during substrate turnover. Their translational diffusivity is generally governed by their size (hydrodynamic radius), shape, and the viscosity of the surrounding medium. It has been argued that the heat of reaction during catalysis affects the structural integrity and internal degrees of freedom of the enzyme. Consequently, this affects the enzyme's performance in a feedback‐loop fashion [108, 109, 110]. Because enzymes exhibit rapid rotational diffusion, each catalytic turnover, occurring at rates between 10 and 10 000 s^−^^1^, results in directional “steps” that change continuously, making the overall movement resemble a random walk, as shown by Berg's foundational work “Random Walks in Biology” [111]. In this regard, Ma et al. reported an increase in the diffusion constant of more than 30% of catalase, urease, and GOx when immobilized on silica NPs (400 nm diameter) [112]. Further, Illien and coworkers employed fluorescence correlation spectroscopy (FCS) to measure the substrate‐dependent diffusion constant of the endothermic and kinetically sluggish enzyme aldolase. Notably, they found that aldolase exhibited a 30% increase in its diffusion constant at saturating substrate concentrations, even in the presence of its inhibitor [108]. In addition, it was reported by Zhang and co‐workers that a 3D‐arranged three‐enzyme assembly of AGO/GOx/HRP on DNA tetrahedra exhibited an accelerated diffusion rate of the assembled enzymes compared to freely floating enzymes in the solution. The authors claimed that this was a contributing player to the overall enhanced catalytic activity of the system, although this was not experimentally correlated [56]. Recent work by Patiño and coworkers highlights the diffusivity of DNA origami nanostructures, together with hosted enzymes [113, 114]. Using single‐particle tracking, they demonstrated that the motility of urease‐covered DNA nanorods is governed by a finely tuned interplay between catalytic density and structural anisotropy. Notably, peak propulsion is achieved at ∼25% urease asymmetric end‐coverage. Further, both urease and catalase, tested for mucosal transport, exhibited enhanced diffusion exclusively when immobilized on a DNA rectangular sheet. Notably, enzymatic behavior was highly sensitive to spatial organization: a symmetric distribution of urease enhanced mobility by increasing the surrounding pH and establishing uniform local pH gradients, whereas an asymmetric arrangement of catalase enabled more effective bubble‐driven propulsion of the whole system. These different studies on various systems underscore the enhanced diffusion of active enzymes. However, Hess argued that the extent of this enhancement should be carefully assessed [115], given that the diffusion constant of active enzymes associated with the translational random steps can be approximated by multiplying the square of the step size by the catalytic rate, yielding added diffusion values below 1 µm^2^/s. This extra theoretical diffusion constant falls in the range of measurement uncertainties, which adds experimental challenges during measurements and data collection [115]. Taken together, these findings suggest that enzyme diffusivity during catalysis contributes not only to the observed enhancement in catalytic performance but also serves as a critical parameter in the rational design of enzyme‐powered nanomotors.

All in all, a plethora of factors contribute to the modulation of enzymatic activity upon immobilization and conjugation to DNA nanostructures. These include the enzymes and DNA nanostructures themselves, but also any modifications, such as fluorophores, mode of attachment, control over the orientation of encapsulated enzymes, as well as the overall experimental set‐up. In order to truly decipher the factors governing the influence of DNA nanostructures on enzymatic activity, all of these factors need to be carefully taken into consideration in future experiments.

## 5 Potential Applications

While the mechanisms underlying the altered behavior of enzymes upon coupling to DNA nanostructures are not yet comprehensively unified, they have been progressively elucidated over the past decade. A substantial body of work has identified key contributing factors and design elements, which enable the employment of DNA–enzyme hybrid nanostructures as candidates for biosensing, biocatalysis, single‐molecule studies, and synthetic nanomachines, which cannot be realized with conventional chemistry techniques. In the following section, we will discuss some of the recent applications of such systems.

**FIGURE 5:**
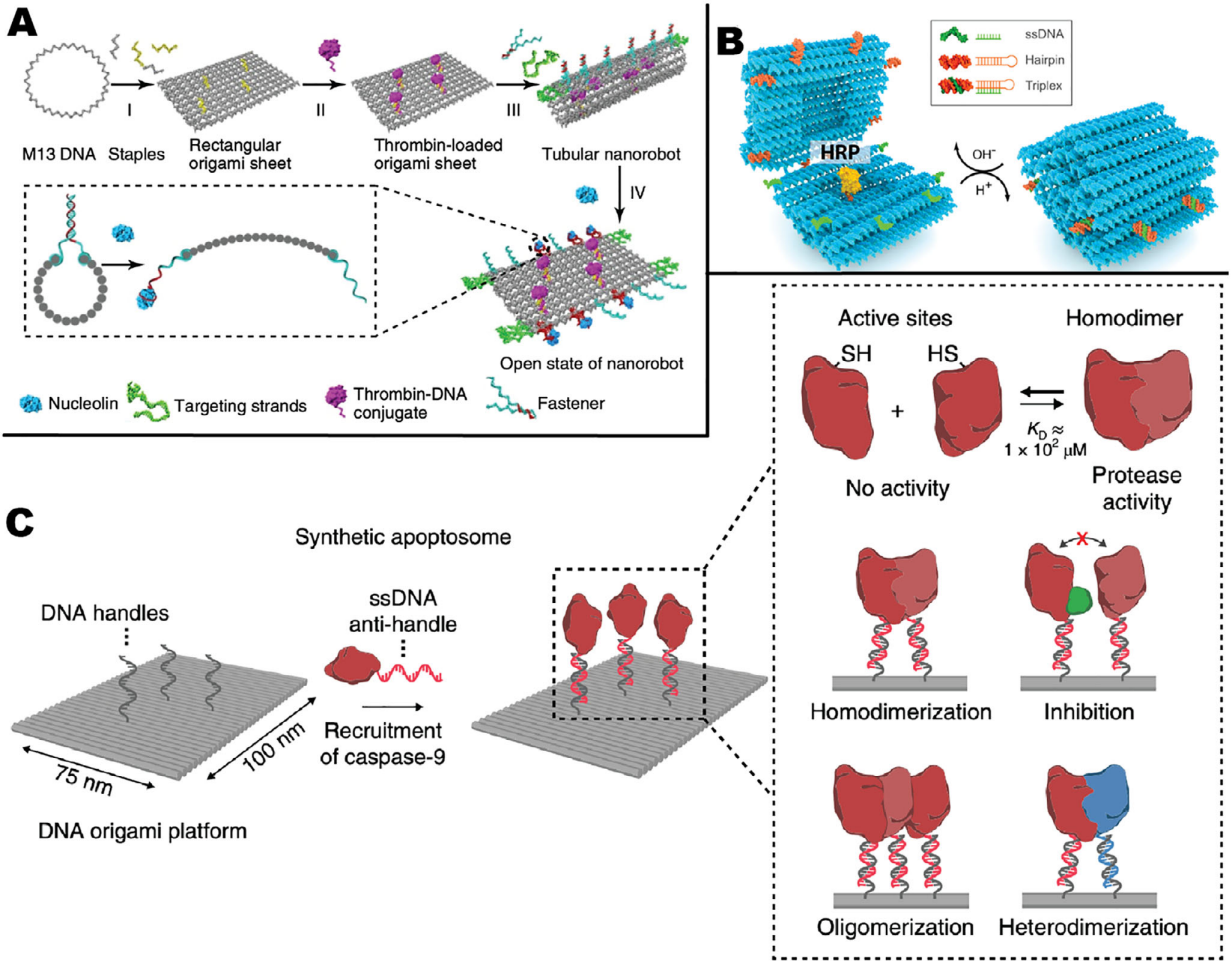
Enzyme–DNA nanostructure applications in targeted delivery, responsive control, and protein interaction studies: (A) Thrombin‐loaded DNA nanorobot: a tubular DNA origami nanostructure, locked by fastener strands and functionalized with targeting aptamers, opens upon nucleolin binding to release thrombin at tumor sites [116]. (B) pH‐responsive DNA capsule: DNA origami nanostructures with triplex motifs reversibly encapsulate and release enzymes (e.g., HRP) in response to pH changes [36]. (C) Synthetic apoptosome platform: Cas9 enzymes are recruited to a DNA origami scaffold via ssDNA handles, enabling controlled dimerization and interaction studies. Various configurations illustrate the effects of spatial organization on activity, inhibition, and dimerization behavior [44]. (A) Adapted with permission [117]. Copyright 2018, Springer Nature. (B) Reproduced with permission [36]. Copyright 2019, ACS. (C) Adapted with permission [44] Copyright 2020, Springer Nature.

Due to the high biocompatibility of DNA nanostructures, DNA–enzyme hybrid systems have been proposed as therapeutic agents. E.g., self‐assembled DNA nanotubes were decorated on the outside with nucleolin‐binding DNA aptamers targeting over‐expressed nucleolin–endothelial cells [116]. The inner cavity of the DNA nanotube was loaded with the blood coagulation protease thrombin. In the presence of its target nucleolin receptor, expressed on the surface of active tumor vascular endothelial cells, the aptamer lock on the DNA nanotubes would open, releasing the encapsulated thrombin at the tumor site (Figure 5A). In in vivo tumor‐bearing models, the DNA–enzyme hybrid system was able to induce intravascular thrombosis and inhibit tumor growth. Importantly, it also did not invoke an immune response in both mice and Bama miniature pigs [116]. Other DNA–enzyme hybrid systems used for targeted delivery included lysozymes [117], CRISPR‐Cas9 [76], HRP and the universal gene modulator catabolite activator protein (CAP) [118], and different DNA nanostructures like rectangular [76], barrel [76]‐shape DNA origami and DNA nanocages [36, 118] with various release mechanisms including temperature [118], and local pH [36]. In an attempt to design a pH‐responsive drug carrier, Linko and co‐workers presented a reconfigurable DNA nanocapsule capable of switching between open and closed states via hairpin/DNA triplexes positioned at the seam of each half of the capsule [36]. At low pH, the hairpin and the corresponding ssDNA are detached, leaving the two halves separated. At higher pH, the ssDNA binds to the hairpin, forming a parallel triplex strand system and locking the capsule in the closed state [36] (Figure 5B). Interestingly, the optimum pH for triggering the Hoogsteen triplex bonding could be manipulated through the sequence of the key ssDNA and percentage of T‐A‐T triplets [119, 120]. Triplex formation/dissociation is characterized by very fast dynamics, which were reported in the opening of the described HRP nanocapsule [36, 119, 120]. However, the closing mechanism described herein required several hours [36].

Further to therapeutic applications, DNA–enzyme hybrid nanostructures provide a flexible and versatile platform to enable mechanistic studies of the function and behavior of many single and multi‐enzyme complexes involved in various cellular processes such as inflammation, innate immunity, and apoptosis ‐among others‐ in controlled environments. One example features a DNA origami‐ enzyme hybrid system mimicking the apoptosome by co‐localizing various caspase‐9 (Cas9) monomers [44]. The platform offered fundamental insights into enzyme clustering and proximity‐induced activation. Both wild‐type and inactive Cas9 were arranged on a planar rectangular DNA origami to enable dimerization or oligomerization of the enzymes [44]. By tethering Cas9 enzymes in close proximity on the DNA sheet, the study demonstrated that dimerization of the enzymes is required for activation. At 6 nm enzyme separation, Cas9 activation resulted in a 23‐fold increase in activation, and it also made the active enzyme dimer 60‐fold resistant to inhibition [44]. In addition, higher Cas9 activity was recorded when more enzymes were clustered together, mimicking multivalent higher‐order enzyme assembly seen in natural signaling complexes. The authors were also able to demonstrate that Cas9 dimerizes asymmetrically, since a heterodimer of wild‐type monomer and an active‐site mutant exhibited similar activity to the wild‐type homodimer [44] (Figure 5C).

DNA‐enzyme hybrid systems have also been applied in synthetic biology to mimic natural systems like the proteasome [20]. For this, Saccà and co‐workers confined two different enzymes to two connected DNA origami nanocages: the first hosted p97, an unfoldase, and the other the protease aCt. p97 was unidirectionally immobilized, allowing target substrates to be recruited, unfolded, released toward the second compartment, then finally degraded by aCt. The fully assembled system had sixfold reduced off‐target degradation [20]. In another study, synthetic DNA nanostructures functionalized with urease or catalase were used to build self‐propelled “DNA–enzyme swimmers” that move autonomously in solution by converting chemical substrates into product gradients [121]. The authors demonstrate fuel‐dependent motility and show that motion can be programmably stopped using a DNA strand–displacement “brake” that removes the enzyme from the scaffold [121]. Many more studies utilized the power of DNA‐enzyme hybrid nanostructures to establish synthetic nanomachines for transcription [18, 122], stepwise biocatalysis [123], and optically‐activated enzymatic gene modification [46].

Another important field where DNA‐enzyme hybrid nanostructures have added value is biosensing and the detection of biomolecules. In this regard, a 3D tetrahedral DNA nanostructure was employed to detect creatinine, which is highly relevant in kidney diseases, at a concentration of 21.4 µM within 90 min [56]. For this purpose, amidohydrolase, creatinase, and sarcosine oxidase (CNH/CRH/SOx) were tethered in equidistance on the edges of the tetrahedron, and HRP was used to correlate the released H_2_O_2_ to the initial creatinine concentration [56]. The reported limit of detection was found to be 10^6^ orders of magnitude higher than current existing methods for creatinine detection [124]. Furthermore, Ricci and coworkers introduced Enzyme‐Linked DNA Displacement (ELIDIS), a two‐step electrochemical assay that achieves ultrasensitive antibody detection by coupling DNA strand displacement with enzymatic amplification [125]. The method uses antigen–DNA conjugates that, upon bivalent binding of a target antibody, trigger a strand‐displacement reaction that releases a GOx–DNA hybrid strand. This enzyme–DNA output strand then hybridizes to a complementary probe on a disposable electrode, producing a quantifiable current proportional to antibody concentration. ELIDIS reaches picomolar limits of detection, works effectively in serum, and is easily adaptable and multiplexable, enabling detection of diverse antibodies—including peptide‐binding antibodies, monoclonal antibodies such as Cetuximab, and even bispecific antibodies—by simply changing the antigen [125]. Another study by Yang et al. deployed the DNAzyme, G‐quadruplex‐hemin (GDH), on a rectangular DNA origami for the detection and quantification of Human Papillomavirus (HPV)‐16 DNA fragments. The HPV‐16 fragment induced crowding of the GDH, which triggered a change in GDH activity, with sensitivity reported down to 4.7 fM. This model serves as a general platform that could be used for other disease detection mechanisms by changing the DNA fragment [84].

## 6 Conclusion

DNA nanotechnology enables the precise construction of predefined 2D and 3D nanostructures, offering sophisticated platforms for biomolecular assembly. This approach allows unprecedented control over enzyme positioning, spacing, and stoichiometry on DNA scaffolds, enabling the manipulation of enzymatic systems. In this review, we introduced the different hypotheses postulated to explain observed effects of enzymatic activity changes upon conjugation to a DNA nanostructure and critically discussed the open gaps in knowledge that remain. We also briefly discussed potential applications of DNA‐enzyme hybrid nanosystems.

Enhanced catalytic activity of many different individual enzymes on DNA nanostructures is a well‐documented phenomenon, though previously proposed mechanisms—including proximity, electrostatic substrate interactions, and localized pH effects—fail to fully explain this phenomenon. Protection against enzyme deactivation, particularly through reduced surface adsorption, contributes to maintaining catalytic activity, but cannot account for the observed enhancements. The hypothesis that an ordered hydration layer near the negatively charged DNA surface stabilizes bound enzymes remains mechanistically unclear and warrants further investigation.

For multi‐enzyme systems, tethering enzymes to DNA nanostructures plays a critical role in modulating catalytic performance. Although spatial proximity between enzymes was initially thought to drive enhanced cascade efficiency, kinetic analyses reveal that this proximity effect becomes negligible in systems with unbalanced enzyme kinetics. The DNA nano‐confined environment reportedly increases local substrate concentrations, potentially enhancing cascade reactions, though the molecular basis for this confinement effect remains undefined.

While mechanistically, many open questions remain, there is without a doubt that DNA‐enzyme hybrid nanosystems possess great potential for a wide variety of applications, from basic research to translational biomedicine. Eventually, a deeper mechanistic understanding of the effects a DNA nanostructure has on the enzymatic activity will advance the design of efficient biocatalytic systems and facilitate the development of sophisticated in vitro metabolic networks, with implications extending beyond DNA‐based nanostructures to other artificial systems.

## Conflicts of Interest

The authors declare no conflicts of interest.

## Data Availability

Data sharing not applicable to this article as no datasets were generated or analysed during the current study.

## References

[1] J. Kim , J. W. Grate , and P. Wang , “Nanostructures for Enzyme Stabilization,” Chemical Engineering Science 61, no. 3 (2006): 1017–1026, 10.1016/j.ces.2005.05.067.

[2] R. A. Sheldon and S. van Pelt , “Enzyme Immobilisation in Biocatalysis: Why, What and How,” Chemical Society Reviews 42, no. 15 (2013): 6223–6235, 10.1039/C3CS60075K. 23532151

[3] Z. Zhou and M. Hartmann , “Progress in Enzyme Immobilization in Ordered Mesoporous Materials and Related Applications,” Chemical Society Reviews 42, no. 9 (2013): 3894, 10.1039/C3CS60059A. 23570038

[4] S. Zhang , J. Bai , W. Kong , et al., “Dendritic Mesoporous Silica Nanoparticles for Enzyme Immobilization,” Green Chemical Engineering 5, no. 2 (2024): 173–186, 10.1016/j.gce.2023.07.002.

[5] J. Zhang , J. F. Lovell , J. Shi , and Y. Zhang , “Nanomaterials for Co‐Immobilization of Multiple Enzymes,” BMEMat 3, no. 1 (2025): 12080, 10.1002/bmm2.12080.

[6] S. Ding , A. A. Cargill , I. L. Medintz , and J. C. Claussen , “Increasing the Activity of Immobilized Enzymes With Nanoparticle Conjugation,” Current Opinion in Biotechnology 34 (2015): 242–250, 10.1016/j.copbio.2015.04.005. 25957941

[7] G. A. Ellis , S. N. Dean , S. A. Walper , and I. L. Medintz , “Quantum Dots and Gold Nanoparticles as Scaffolds for Enzymatic Enhancement: Recent Advances and the Influence of Nanoparticle Size,” Catalysts 10, no. 1 (2020): 83, 10.3390/catal10010083.

[8] K. Ponmudi , A. R. Cherian , and A. Varghese , “Carbon Dots as an Effective Material in Enzyme Immobilization for Sensing Applications,” in Carbon Dots in Analytical Chemistry, ed. S. K. Kailasa and C. M. Hussain (Elsevier, 2023), 241–253, 10.1016/B978-0-323-98350-1.00006-2.

[9] W. Feng and P. Ji , “Enzymes Immobilized on Carbon Nanotubes,” Biotechnology Advances 29, no. 6 (2011): 889–895, 10.1016/j.biotechadv.2011.07.007. 21820044

[10] Y. R. Maghraby , R. M. El‐Shabasy , A. H. Ibrahim , and H. M. E.‐S. Azzazy , “Enzyme Immobilization Technologies and Industrial Applications,” ACS Omega 8, no. 6 (2023): 5184–5196, 10.1021/acsomega.2c07560. 36816672 PMC9933091

[11] X. Lyu , R. Gonzalez , A. Horton , and T. Li , “Immobilization of Enzymes by Polymeric Materials,” Catalysts 11, no. 10 (2021): 1211, 10.3390/catal11101211.

[12] X. Lian , Y. Fang , E. Joseph , et al., “Enzyme–MOF (Metal–Organic Framework) Composites,” Chemical Society Reviews 46, no. 11 (2017): 3386–3401, 10.1039/C7CS00058H. 28451673

[13] Y. Su , B. Liu , Z. Huang , et al., “Virus‐Like Particles Nanoreactors: From Catalysis towards Bio‐Applications,” Journal of Materials Chemistry B 11, no. 38 (2023): 9084–9098, 10.1039/D3TB01112G. 37697810

[14] N. C. Seeman , “Nucleic Acid Junctions and Lattices,” Journal of Theoretical Biology 99, no. 2 (1982): 237–247, 10.1016/0022-5193(82)90002-9. 6188926

[15] S. Dey , C. Fan , K. V. Gothelf , et al., “DNA Origami,” Nature Reviews Methods Primers 1, no. 1 (2021): 13, 10.1038/s43586-020-00009-8.

[16] P. W. K. Rothemund , “Folding DNA to Create Nanoscale Shapes and Patterns,” Nature 440, no. 7082 (2006): 297–302, 10.1038/nature04586. 16541064

[17] C. Kielar , Y. Xin , X. Xu , et al., “Effect of Staple Age on DNA Origami Nanostructure Assembly and Stability,” Molecules 24, no. 14 (2019): 2577, 10.3390/molecules24142577. 31315177 PMC6680526

[18] S. F. J. Wickham , A. Auer , J. Min , et al., “Complex Multicomponent Patterns Rendered on a 3D DNA‐Barrel Pegboard,” Nature Communications 11, no. 1 (2020): 5768, 10.1038/s41467-020-18910-x. PMC7666213 33188187

[19] H. Dietz , S. M. Douglas , and W. M. Shih , “Folding DNA into Twisted and Curved Nanoscale Shapes,” Science 325, no. 5941 (2009): 725–730, 10.1126/science.1174251. 19661424 PMC2737683

[20] J. Huang , A. Jaekel , J. van den Boom , et al., “A Modular DNA Origami Nanocompartment for Engineering a Cell‐Free, Protein Unfolding and Degradation Pathway,” Nature Nanotechnology 19 (2024): 1521–1531, 10.1038/s41565-024-01738-7. PMC11486656 39075293

[21] J. M. Weck and A. Heuer‐Jungemann , “Fully Addressable Designer Superstructures Assembled From One Single Modular DNA Origami,” Nature Communications 16, no. 1 (2025): 1556, 10.1038/s41467-025-56846-2. PMC11814417 39934172

[22] J. J. Schmied , M. Raab , C. Forthmann , et al., “DNA Origami–Based Standards for Quantitative Fluorescence Microscopy,” Nature Protocols 9, no. 6 (2014): 1367–1391, 10.1038/nprot.2014.079. 24833175

[23] V. Linko , M. Eerikäinen , and M. A. Kostiainen , “A Modular DNA Origami‐Based Enzyme Cascade Nanoreactor,” Chemical Communications 51, no. 25 (2015): 5351–5354, 10.1039/c4cc08472a. 25594847

[24] S. Rudiuk , A. Venancio‐Marques , and D. Baigl , “Enhancement and Modulation of Enzymatic Activity through Higher‐Order Structural Changes of Giant DNA–Protein Multibranch Conjugates,” Angewandte Chemie International Edition 51, no. 51 (2012): 12694–12698, 10.1002/anie.201206962. 23143988

[25] Y. Gao , C. C. Roberts , A. Toop , C.‐E. A. Chang , and I. Wheeldon , “Mechanisms of Enhanced Catalysis in Enzyme–DNA Nanostructures Revealed through Molecular Simulations and Experimental Analysis,” Chembiochem 17, no. 15 (2016): 1430–1436, 10.1002/cbic.201600224. 27173175

[26] Y. Zhang , Q. Wang , and H. Hess , “Increasing Enzyme Cascade Throughput by pH‐Engineering the Microenvironment of Individual Enzymes,” ACS Catalysis 7, no. 3 (2017): 2047–2051, 10.1021/acscatal.6b03431.

[27] S. Lim , J. Kim , Y. Kim , D. Xu , and D. S. Clark , “CRISPR/Cas‐Directed Programmable Assembly of Multi‐Enzyme Complexes,” Chemical Communications 56, no. 36 (2020): 4950–4953, 10.1039/D0CC01174F. 32239050

[28] J. Müller and C. M. Niemeyer , “DNA‐Directed Assembly of Artificial Multienzyme Complexes,” Biochemical and Biophysical Research Communications 377, no. 1 (2008): 62–67, 10.1016/j.bbrc.2008.09.078. 18823945

[29] O. I. Wilner , Y. Weizmann , R. Gill , O. Lioubashevski , R. Freeman , and I. Willner , “Enzyme Cascades Activated on Topologically Programmed DNA Scaffolds,” Nature Nanotechnology 4, no. 4 (2009): 249–254, 10.1038/nnano.2009.50. 19350036

[30] G. Grossi , A. Jaekel , E. S. Andersen , and B. Saccà , “Enzyme‐Functionalized DNA Nanostructures as Tools for Organizing and Controlling Enzymatic Reactions,” MRS Bulletin 42, no. 12 (2017): 920–924, 10.1557/mrs.2017.269.

[31] A. Jaekel , P. Stegemann , and B. Saccà , “Manipulating Enzymes Properties With DNA Nanostructures,” Molecules 24, no. 20 (2019): 3694, 10.3390/molecules24203694. 31615123 PMC6832416

[32] P. Lin , H. Yang , E. Nakata , and T. Morii , “Mechanistic Aspects for the Modulation of Enzyme Reactions on the DNA Scaffold,” Molecules 27, no. 19 (2022): 6309, 10.3390/molecules27196309. 36234845 PMC9572797

[33] K. S. Rabe , J. Müller , M. Skoupi , and C. M. Niemeyer , “Cascades in Compartments: En Route to Machine‐Assisted Biotechnology,” Angewandte Chemie International Edition 56, no. 44 (2017): 13574–13589, 10.1002/anie.201703806. 28691387

[34] S. Kröll and C. M. Niemeyer , “Nucleic Acid‐Based Enzyme Cascades—Current Trends and Future Perspectives,” Angewandte Chemie International Edition 63, no. 5 (2024): 202314452, 10.1002/anie.202314452. 37870888

[35] S. Juul , F. Iacovelli , M. Falconi , et al., “Temperature‐Controlled Encapsulation and Release of an Active Enzyme in the Cavity of a Self‐Assembled DNA Nanocage,” ACS Nano 7, no. 11 (2013): 9724–9734, 10.1021/nn4030543. 24168393

[36] H. Ijäs , I. Hakaste , B. Shen , M. A. Kostiainen , and V. Linko , “Reconfigurable DNA Origami Nanocapsule for pH‐Controlled Encapsulation and Display of Cargo,” ACS Nano 13, no. 5 (2019): 5959–5967, 10.1021/acsnano.9b01857. 30990664 PMC7076726

[37] I. Seitz , D. McNeale , F. Sainsbury , V. Linko , and M. A. Kostiainen , “Modular Virus Capsid Coatings for Biocatalytic DNA Origami Nanoreactors,” ACS Nano 19 (2025): 36465–36477, 10.1021/acsnano.5c10734. 41060700 PMC12548342

[38] G. Grossi , M. Dalgaard Ebbesen Jepsen , J. Kjems , and E. S. Andersen , “Control of Enzyme Reactions by a Reconfigurable DNA Nanovault,” Nature Communications 8, no. 1 (2017): 992, 10.1038/s41467-017-01072-8. PMC5648847 29051565

[39] H. Dinh , E. Nakata , K. Mutsuda‐Zapater , M. Saimura , M. Kinoshita , and T. Morii , “Enhanced Enzymatic Activity Exerted by a Packed Assembly of a Single Type of Enzyme,” Chemical Science 11, no. 34 (2020): 9088–9100, 10.1039/d0sc03498c. 34094190 PMC8161546

[40] P. Lin , T. Hayashi , H. Dinh , E. Nakata , M. Kinoshita , and T. Morii , “Enzyme Reactions Are Accelerated or Decelerated When the Enzymes Are Located near the DNA Nanostructure,” ACS Applied Materials & Interfaces 17, no. 10 (2025): 15775–15792, 10.1021/acsami.4c18192. 40075560 PMC11912197

[41] R. Kosinski , J. M. Perez , E.‐C. Schöneweiß , et al., “The Role of DNA Nanostructures in the Catalytic Properties of an Allosterically Regulated Protease,” Science Advances 8, no. 1 (2022): abk0425, 10.1126/sciadv.abk0425. PMC8730604 34985948

[42] P. Lin , H. Dinh , E. Nakata , and T. Morii , “Dynamic Shape Transformation of a DNA Scaffold Applied for an Enzyme Nanocarrier,” Frontiers in Chemistry 9, no. 453 (2021): 697857, 10.3389/fchem.2021.697857. 34249866 PMC8263910

[43] J. Huang , A. Suma , M. Cui , et al., “Arranging Small Molecules With Subnanometer Precision on DNA Origami Substrates for the Single‐Molecule Investigation of Protein–Ligand Interactions,” Small Structures 1, no. 1 (2020): 2000038, 10.1002/sstr.202000038.

[44] B. J. H. M. Rosier , A. J. Markvoort , G. Audenis , et al., “Proximity‐Induced Caspase‐9 Activation on a DNA Origami‐Based Synthetic Apoptosome,” Nature Catalysis 3, no. 3 (2020): 295–306, 10.1038/s41929-019-0403-7. PMC7080557 32190819

[45] E. Weinhold and B. Chakraborty , “DNA Modification and Visualization on an Origami‐Based Enzyme Nano‐Factory,” Nanoscale 13, no. 4 (2021): 2465–2471, 10.1039/d0nr07618j. 33471009

[46] K. Abe , H. Sugiyama , and M. Endo , “Construction of an Optically Controllable CRISPR‐Cas9 System Using a DNA Origami Nanostructure,” Chemical Communications 57, no. 45 (2021): 5594–5596, 10.1039/D1CC00876E. 33982688

[47] Z. Zhao , J. Fu , S. Dhakal , et al., “Nanocaged Enzymes With Enhanced Catalytic Activity and Increased Stability Against Protease Digestion,” Nature Communications 7, no. 1 (2016): 10619, 10.1038/ncomms10619. PMC4749968 26861509

[48] H. Yang , P. Lin , S. Chuaychob , et al., “Construction of a CO_2_‐Fixing Compartment Using a Shape‐Transforming DNA Scaffold,” Chemistry – A European Journal (2026): 03305, 10.1002/chem.202503305. PMC13206248 41766453

[49] J. Fu , M. Liu , Y. Liu , N. W. Woodbury , and H. Yan , “Interenzyme Substrate Diffusion for an Enzyme Cascade Organized on Spatially Addressable DNA Nanostructures,” Journal of the American Chemical Society 134, no. 12 (2012): 5516–5519, 10.1021/ja300897h. 22414276 PMC3319985

[50] Y. Fu , D. Zeng , J. Chao , et al., “Single‐Step Rapid Assembly of DNA Origami Nanostructures for Addressable Nanoscale Bioreactors,” Journal of the American Chemical Society 135, no. 2 (2013): 696–702, 10.1021/ja3076692. 23237536

[51] J. S. Kahn , Y. Xiong , J. Huang , and O. Gang , “Cascaded Enzyme Reactions Over a Three‐Dimensional, Wireframe DNA Origami Scaffold,” JACS Au 2 (2022): 357–366, 10.1021/jacsau.1c00387. 35252986 PMC8889550

[52] Y. Zhang , S. Tsitkov , and H. Hess , “Proximity Does Not Contribute to Activity Enhancement in the Glucose Oxidase–Horseradish Peroxidase Cascade,” Nature Communications 7, no. 1 (2016): 13982, 10.1038/ncomms13982. PMC5196434 28004753

[53] T. A. Ngo , E. Nakata , M. Saimura , and T. Morii , “Spatially Organized Enzymes Drive Cofactor‐Coupled Cascade Reactions,” Journal of the American Chemical Society 138, no. 9 (2016): 3012–3021, 10.1021/jacs.5b10198. 26881296

[54] J. Fu , Y. R. Yang , A. Johnson‐Buck , et al., “Multi‐Enzyme Complexes on DNA Scaffolds Capable of Substrate Channelling With an Artificial Swinging Arm,” Nature Nanotechnology 9, no. 7 (2014): 531–536, 10.1038/nnano.2014.100. 24859813

[55] M. Liu , J. Fu , X. Qi , et al., “A Three‐Enzyme Pathway With an Optimised Geometric Arrangement to Facilitate Substrate Transfer,” Chembiochem 17, no. 12 (2016): 1097–1101, 10.1002/cbic.201600103. 26995014

[56] N. Cao , R. Guo , P. Song , et al., “DNA Framework–Programmed Nanoscale Enzyme Assemblies,” Nano Letters 24, no. 15 (2024): 4682–4690, 10.1021/acs.nanolett.4c01137. 38563501

[57] G. Ke , M. Liu , S. Jiang , et al., “Directional Regulation of Enzyme Pathways Through the Control of Substrate Channeling on a DNA Origami Scaffold,” Angewandte Chemie International Edition 55, no. 26 (2016): 7483–7486, 10.1002/anie.201603183. 27159899

[58] H. Dinh , E. Nakata , P. Lin , M. Saimura , H. Ashida , and T. Morii , “Reaction of Ribulose Biphosphate Carboxylase/Oxygenase Assembled on a DNA Scaffold,” Bioorganic & Medicinal Chemistry 27, no. 22 (2019): 115120, 10.1016/j.bmc.2019.115120. 31627975

[59] S. Kröll , K. S. Rabe , and C. M. Niemeyer , “An Orthogonal Covalent Connector System for the Efficient Assembly of Enzyme Cascades on DNA Nanostructures,” Small 17 (2021): 2105095, 10.1002/smll.202105095. 34825457

[60] P. Lin , H. Dinh , Y. Morita , et al., “Evaluation of the Role of the DNA Surface for Enhancing the Activity of Scaffolded Enzymes,” Chemical Communications 57, no. 32 (2021): 3925–3928, 10.1039/D1CC00276G. 33871490

[61] P. Lin , H. Dinh , E. Nakata , and T. Morii , “Conditional Dependence of Enzyme Cascade Reaction Efficiency on the Inter‐Enzyme Distance,” Chemical Communications 57, no. 85 (2021): 11197–11200, 10.1039/D1CC04162B. 34622899

[62] Z. Wang , E. S. Iago‐Mcrae A. Ebrahimimojarad , S. W. Oh , and J. Fu , “Modulation of Enzyme Cascade Activity by Local Substrate Enrichment and Exclusion on DNA Nanostructures,” Langmuir 38 (2022): 12594–12601, 10.1021/acs.langmuir.2c02064. 36194827

[63] S. Kröll , T. Burgahn , K. S. Rabe , M. Franzreb , and C. M. Niemeyer , “Nano‐ and Microscale Confinements in DNA‐Scaffolded Enzyme Cascade Reactions,” Small 20, no. 4 (2024): 2304578, 10.1002/smll.202304578. 37732702

[64] W. P. Klein , R. P. Thomsen , K. B. Turner , et al., “Enhanced Catalysis From Multienzyme Cascades Assembled on a DNA Origami Triangle,” ACS Nano 13, no. 12 (2019): 13677–13689, 10.1021/acsnano.9b05746. 31751123

[65] M. Madsen and K. V. Gothelf , “Chemistries for DNA Nanotechnology,” Chemical Reviews 119, no. 10 (2019): 6384–6458, 10.1021/acs.chemrev.8b00570. 30714731

[66] L. M. Van Der Sleen and K. M. Tych , “Bioconjugation Strategies for Connecting Proteins to DNA‐Linkers for Single‐Molecule Force‐Based Experiments,” Nanomaterials 11, no. 9 (2021): 2424, 10.3390/nano11092424. 34578744 PMC8464727

[67] M. Budiarta , M. Streit , and G. Beliu , “Site‐Specific Protein Labeling Strategies for Super‐Resolution Microscopy,” Current Opinion in Chemical Biology 80 (2024): 102445, 10.1016/j.cbpa.2024.102445. 38490137

[68] D. Zhao , Y. Kong , S. Zhao , and H. Xing , “Engineering Functional DNA–Protein Conjugates for Biosensing, Biomedical, and Nanoassembly Applications,” Topics in Current Chemistry 378, no. 3 (2020): 41, 10.1007/s41061-020-00305-7. 32447526

[69] I. Murphy , K. Bobilev , D. Hayakawa , et al., “A Method for Site‐Specifically Tethering the Enzyme Urease to DNA Origami With Sustained Activity,” PLoS ONE 20, no. 4 (2025): 0319790, 10.1371/journal.pone.0319790. PMC12011258 40258063

[70] A. Tantipanjaporn and M. K. Wong , “Development and Recent Advances in Lysine and N‐Terminal Bioconjugation for Peptides and Proteins,” Molecules 28, no. 3 (2023): 1083, 10.3390/molecules28031083. 36770752 PMC9953373

[71] Y. R. Yang , J. Fu , S. Wootten , et al., “2D Enzyme Cascade Network With Efficient Substrate Channeling by Swinging Arms,” Chembiochem 19, no. 3 (2018): 212–216, 10.1002/cbic.201700613. 29178416

[72] R. M. L. Berger , J. M. Weck , S. M. Kempe , et al., “Nanoscale FasL Organization on DNA Origami to Decipher Apoptosis Signal Activation in Cells,” Small 17, no. 26 (2021): 2101678, 10.1002/smll.202101678. 34057291

[73] J. M. Weck , R. Nair , M.‐Z. Kesici , et al., “Effects of DNA Origami‐Based Nanoagent Design on Apoptosis Induction in a Large 3D Cancer Spheroid Model,” Small 21, no. 24 (2025): 2502490, 10.1002/smll.202502490. 40277317 PMC12177858

[74] S. Kröll , L. Schneider , P. Wadhwani , K. S. Rabe , and C. M. Niemeyer , “Orthogonal Protein Decoration of DNA Nanostructures Based on SpyCatcher–SpyTag Interaction,” Chemical Communications 58, no. 97 (2022): 13471–13474, 10.1039/D2CC05335G. 36383063

[75] E. A. Berckman and W. Chen , “Exploiting dCas9 Fusion Proteins for Dynamic Assembly of Synthetic Metabolons,” Chemical Communications 55, no. 57 (2019): 8219–8222, 10.1039/C9CC04002A. 31210215 PMC7725109

[76] J. Yang , X. Xu , L. Yang , Y. Tian , J. Wang , and D. Han , “Dynamic Genomic Imaging and Tracking in Living Cells by a DNA Origami‐Based CRISPR‒dCas9 System,” Small Methods 9 (2025): 2401559, 10.1002/smtd.202401559. 39828625

[77] H. C. Kolb , M. G. Finn , and K. B. Sharpless , “Click Chemistry: Diverse Chemical Function From a Few Good Reactions,” Angewandte Chemie International Edition 40, no. 11 (2001): 2004–2021, 10.1002/1521-3773(20010601)40:11. 11433435

[78] N. Li and W. H. Binder , “Click‐Chemistry for Nanoparticle‐Modification,” Journal of Materials Chemistry 21, no. 42 (2011): 16717, 10.1039/C1JM11558H.

[79] B. Stump , “Click Bioconjugation: Modifying Proteins Using Click‐like Chemistry,” Chembiochem 23, no. 16 (2022): 202200016, 10.1002/cbic.202200016. 35491526

[80] N. K. Devaraj and M. G. Finn , “Introduction: Click Chemistry,” Chemical Reviews 121, no. 12 (2021): 6697–6698, 10.1021/acs.chemrev.1c00469. 34157843

[81] L. Wang , A. Brock , B. Herberich , and P. G. Schultz , “Expanding the Genetic Code of *Escherichia coli* ,” Science 292, no. 5516 (2001): 498–500, 10.1126/science.1060077. 11313494

[82] L. E. Wilkins , M. Hasan , A. E. R. Fayter , C. Biggs , M. Walker , and M. I. Gibson , “Site‐Specific Conjugation of Antifreeze Proteins onto Polymer‐Stabilized Nanoparticles,” Polymer Chemistry 10, no. 23 (2019): 2986–2990, 10.1039/C8PY01719K. 31303900 PMC6592154

[83] Z. Ge , J. Fu , M. Liu , et al., “Constructing Submonolayer DNA Origami Scaffold on Gold Electrode for Wiring of Redox Enzymatic Cascade Pathways,” ACS Applied Materials & Interfaces 11, no. 15 (2019): 13881–13887, 10.1021/acsami.8b12374. 30379533

[84] B. Yang , R. Wang , W. Li , J. Wang , and H. Liu , “On‐Origami Molecular Crowding Control of G‐Quadruplex DNAzymes,” Small Methods 9 (2025): 2401401, 10.1002/smtd.202401401. 39935188

[85] M. Sutter , T. G. Laughlin , N. B. Sloan , D. Serwas , K. M. Davies , and C. A. Kerfeld , “Structure of a Synthetic β‐Carboxysome Shell,” Plant Physiology 181, no. 3 (2019): 1050–1058, 10.1104/pp.19.00885. 31501298 PMC6836842

[86] W. Bonacci , P. K. Teng , B. Afonso , et al., “Modularity of a Carbon‐Fixing Protein Organelle,” Proceedings of the National Academy of Sciences 109, no. 2 (2012): 478–483, 10.1073/pnas.1108557109. PMC3258634 22184212

[87] R. Satange and M. H. Hou , “The Role of Water in Mediating DNA Structures With Epigenetic Modifications, Higher‐Order Conformations and Drug–DNA Interactions,” RSC Chemical Biology 6, no. 5 (2025): 699–720, 10.1039/d4cb00308j. 40171245 PMC11955920

[88] S. Ebbinghaus , S. J. Kim , M. Heyden , et al., “An Extended Dynamical Hydration Shell around Proteins,” Proceedings of the National Academy of Sciences 104, no. 52 (2007): 20749–20752, 10.1073/pnas.0709207104. PMC2410073 18093918

[89] M. L. McDermott , H. Vanselous , S. A. Corcelli , and P. B. Petersen , “DNA's Chiral Spine of Hydration,” ACS Central Science 3, no. 7 (2017): 708–714, 10.1021/acscentsci.7b00100. 28776012 PMC5532714

[90] S. Nakano , D. Yamaguchi , H. Tateishi‐Karimata , D. Miyoshi , and N. Sugimoto , “Hydration Changes Upon DNA Folding Studied by Osmotic Stress Experiments,” Biophysical Journal 102, no. 12 (2012): 2808–2817, 10.1016/j.bpj.2012.05.019. 22735531 PMC3379618

[91] A. Zinchenko , N. V. Berezhnoy , S. Wang , et al., “Single‐Molecule Compaction of Megabase‐Long Chromatin Molecules by Multivalent Cations,” Nucleic Acids Research 46, no. 2 (2017): 635–649, 10.1093/nar/gkx1135. PMC5778610 29145649

[92] A. Kuhn , C. V. Zyl , A. V. Tonder , and B. A. Prior , “Purification and Partial Characterization of an Aldo‐Keto Reductase From Saccharomyces Cerevisiae,” Applied and Environmental Microbiology 61, no. 4 (1995): 1580–1585, 10.1128/aem.61.4.1580-1585.1995. 7747971 PMC167412

[93] N. H. Schlieben , K. Niefind , J. Müller , B. Riebel , W. Hummel , and D. Schomburg , “Atomic Resolution Structures of R‐Specific Alcohol Dehydrogenase From Lactobacillus Brevis Provide the Structural Bases of Its Substrate and Cosubstrate Specificity,” Journal of Molecular Biology 349, no. 4 (2005): 801–813, 10.1016/j.jmb.2005.04.029. 15896805

[94] E. Fossati , F. Polentini , G. Carrea , and S. Riva , “Exploitation of the Alcohol Dehydrogenase‐Acetone NADP‐Regeneration System for the Enzymatic Preparative‐Scale Production of 12‐Ketochenodeoxycholic Acid,” Biotechnology and Bioengineering 93, no. 6 (2006): 1216–1220, 10.1002/bit.20753. 16245351

[95] A. A. Almaqwashi , T. Paramanathan , I. Rouzina , and M. C. Williams , “Mechanisms of Small Molecule–DNA Interactions Probed by Single‐Molecule Force Spectroscopy,” Nucleic Acids Research 44, no. 9 (2016): 3971–3988, 10.1093/nar/gkw237. 27085806 PMC4872107

[96] Y. Gao , C. C. Roberts , J. Zhu , J.‐L. Lin , C. Chang , and I. Wheeldon , “Tuning Enzyme Kinetics through Designed Intermolecular Interactions Far From the Active Site,” ACS Catalysis 5, no. 4 (2015): 2149–2153, 10.1021/acscatal.5b00130.

[97] J.‐L. Lin and I. Wheeldon , “Kinetic Enhancements in DNA–Enzyme Nanostructures Mimic the Sabatier Principle,” ACS Catalysis 3, no. 4 (2013): 560–564, 10.1021/cs300766d.

[98] Q. Li , Z. Armstrong , A. MacRae , M. Lenertz , L. Feng , and Z. Yang , “On the Interface of Enzyme and Spatial Confinement: The Impacts of Confinement Rigidity, Shape, and Surface Properties on the Interplay of Enzyme Structure, Dynamics, and Function,” Chemical Physics Reviews 4, no. 4 (2023): 041302, 10.1063/5.0167117.

[99] I. J. Minten , V. I. Claessen , K. Blank , A. E. Rowan , R. J. M. Nolte , and J. J. L. M. Cornelissen , “Catalytic Capsids: The Art of Confinement,” Chemical Science 2, no. 2 (2011): 358–362, 10.1039/C0SC00407C.

[100] A. Küchler , M. Yoshimoto , S. Luginbühl , F. Mavelli , and P. Walde , “Enzymatic Reactions in Confined Environments,” Nature Nanotechnology 11, no. 5 (2016): 409–420, 10.1038/nnano.2016.54. 27146955

[101] C. Eun , P. M. Kekenes‐Huskey , V. T. Metzger , and J. A. McCammon , “A Model Study of Sequential Enzyme Reactions and Electrostatic Channeling,” The Journal of Chemical Physics 140, no. 10 (2014): 105101, 10.1063/1.4867286. 24628210 PMC3977847

[102] I. Wheeldon , S. D. Minteer , S. Banta , S. C. Barton , P. Atanassov , and M. Sigman , “Substrate Channelling as an Approach to Cascade Reactions,” Nature Chemistry 8, no. 4 (2016): 299–309, 10.1038/nchem.2459. 27001725

[103] S. A. M. van Stroe‐Biezen , F. M. Everaerts , L. J. J. Janssen , and R. A. Tacken , “Diffusion Coefficients of Oxygen, Hydrogen Peroxide and Glucose in a Hydrogel,” Analytica Chimica Acta 273, no. 1 (1993): 553–560, 10.1016/0003-2670(93)80202-V.

[104] L. Sun , Y. Gao , Y. Xu , et al., “Real‐Time Imaging of Single‐Molecule Enzyme Cascade Using a DNA Origami Raft,” Journal of the American Chemical Society 139, no. 48 (2017): 17525–17532, 10.1021/jacs.7b09323. 29131610

[105] A. Bartesaghi , D. Matthies , S. Banerjee , A. Merk , and S. Subramaniam , “Structure of β‐galactosidase at 3.2‐Å Resolution Obtained by Cryo‐Electron Microscopy,” Proceedings of the National Academy of Sciences 111, no. 32 (2014): 11709–11714, 10.1073/pnas.1402809111. PMC4136629 25071206

[106] W. Abdallah , V. Chirino , I. Wheeldon , and S. Banta , “Catalysis of Thermostable Alcohol Dehydrogenase Improved by Engineering the Microenvironment through Fusion With Supercharged Proteins,” Chembiochem 20, no. 14 (2019): 1827–1837, 10.1002/cbic.201900066. 30859665

[107] M. Scherf , F. Scheffler , C. Maffeo , et al., “Trapping of Protein Cargo Molecules inside DNA Origami Nanocages,” Nanoscale 14, no. 48 (2022): 18041–18050, 10.1039/D2NR05356J. 36445741

[108] P. Illien , X. Zhao , K. K. Dey , P. J. Butler , A. Sen , and R. Golestanian , “Exothermicity Is Not a Necessary Condition for Enhanced Diffusion of Enzymes,” Nano Letters 17, no. 7 (2017): 4415–4420, 10.1021/acs.nanolett.7b01502. 28593755

[109] C. Riedel , R. Gabizon , C. A. M. Wilson , et al., “The Heat Released during Catalytic Turnover Enhances the Diffusion of an Enzyme,” Nature 517, no. 7533 (2015): 227–230, 10.1038/nature14043. 25487146 PMC4363105

[110] A.‐Y. Jee , Y.‐K. Cho , S. Granick , and T. Tlusty , “Catalytic Enzymes Are Active Matter,” Proceedings of the National Academy of Sciences 115, no. 46 (2018): E10812–E10821, 10.1073/pnas.1814180115. PMC6243271 30385635

[111] H. C. Berg , Random Walks in Biology (Princeton University Press, 1993).

[112] X. Ma , A. Jannasch , U.‐R. Albrecht , et al., “Enzyme‐Powered Hollow Mesoporous Janus Nanomotors,” Nano Letters 15, no. 10 (2015): 7043–7050, 10.1021/acs.nanolett.5b03100. 26437378

[113] L. Paffen , M. Engelbert van Bevervoorde , A. Rodriguez‐Abetxuko , et al., “Programmable Nanoscale Motion via Molecular Patterning on DNA Origami,” Angewandte Chemie International Edition 65, no. 8 (2026): 23921, 10.1002/anie.202523921. PMC12910145 41546160

[114] M. Tollemeto , E. Tsang , L. J. M. M. Paffen , et al., “Enzyme‐Powered DNA Origami Nanostructures for Enhanced Mucosal Diffusion,” Small Structures 7, no. 3 (2026): 70373, 10.1002/sstr.70373.

[115] Y. Zhang and H. Hess , “Enhanced Diffusion of Catalytically Active Enzymes,” ACS Central Science 5, no. 6 (2019): 939–948, 10.1021/acscentsci.9b00228. 31263753 PMC6598160

[116] S. Li , Q. Jiang , S. Liu , et al., “A DNA Nanorobot Functions as a Cancer Therapeutic in Response to a Molecular Trigger in Vivo,” Nature Biotechnology 36, no. 3 (2018): 258–264, 10.1038/nbt.4071. 29431737

[117] I. Mela , P. P. Vallejo‐Ramirez , S. Makarchuk , et al., “DNA Nanostructures for Targeted Antimicrobial Delivery,” Angewandte Chemie International Edition 59, no. 31 (2020): 12698–12702, 10.1002/anie.202002740. 32297692 PMC7496991

[118] R. Crawford , C. M. Erben , J. Periz , et al., “Non‐Covalent Single Transcription Factor Encapsulation inside a DNA Cage,” Angewandte Chemie International Edition 52, no. 8 (2013): 2284–2288, 10.1002/anie.201207914. 23325751

[119] A. Idili and F. Ricci , “Design and Characterization of pH‐Triggered DNA Nanoswitches and Nanodevices Based on DNA Triplex Structures,” in DNA Nanotechnology: Methods and Protocols, ed. G. Zuccheri (Springer, 2018), 79–100, 10.1007/978-1-4939-8582-1_6. 29926447

[120] Y. Hu , A. Cecconello , A. Idili , F. Ricci , and I. Willner , “Triplex DNA Nanostructures: From Basic Properties to Applications,” Angewandte Chemie International Edition 56, no. 48 (2017): 15210–15233, 10.1002/anie.201701868. 28444822

[121] T. Patiño Padial , E. Del Grosso , S. Gentile , et al., “Synthetic DNA‐Based Swimmers Driven by Enzyme Catalysis,” Journal of the American Chemical Society 146, no. 18 (2024): 12664–12671, 10.1021/jacs.4c02094. 38587543

[122] J. Hahn , L. Y. T. Chou , R. S. Sørensen , R. M. Guerra , and W. M. Shih , “Extrusion of RNA From a DNA‐Origami‐Based Nanofactory,” ACS Nano 14, no. 2 (2020): 1550–1559, 10.1021/acsnano.9b06466. 31922721

[123] Y. Yang , S. Zhang , S. Yao , et al., “Programming Rotary Motions With a Hexagonal DNA Nanomachine,” Chemistry – A European Journal 25, no. 20 (2019): 5158–5162, 10.1002/chem.201900221. 30791173

[124] E. Mohabbati‐Kalejahi , V. Azimirad , M. Bahrami , and A. Ganbari , “A Review on Creatinine Measurement Techniques,” Talanta 97 (2012): 1–8, 10.1016/j.talanta.2012.04.005. 22841040

[125] A. Díaz‐Fernández , S. Ranallo , and F. Ricci , “Enzyme‐Linked DNA Displacement (ELIDIS) Assay for Ultrasensitive Electrochemical Detection of Antibodies**,” Angewandte Chemie International Edition 63, no. 1 (2024): 202314818, 10.1002/anie.202314818. 37994381

